# Developmental Outcomes for Children at High Risk of Dyslexia and Children With Developmental Language Disorder

**DOI:** 10.1111/cdev.13216

**Published:** 2019-01-24

**Authors:** Margaret J. Snowling, Hannah M. Nash, Debbie C. Gooch, Marianna E. Hayiou‐Thomas, Charles Hulme, Wellcome Language and Reading Project Team

**Affiliations:** ^1^ University of Oxford; ^2^ University of Leeds; ^3^ University of Surrey; ^4^ University of York

## Abstract

We followed children at family risk of dyslexia and children with preschool language difficulties from age 3½, comparing them with controls (*N *=* *234). At age 8, children were classified as having dyslexia or Developmental Language Disorder (DLD) and compared at earlier time points with controls. Children with dyslexia have specific difficulties with phonology and emergent reading skills in the preschool period, whereas children with DLD, with or without dyslexia, show a wider range of impairments including significant problems with executive and motor tasks. For children with both dyslexia and DLD, difficulties with phonology are generally more severe than those observed in children with dyslexia or DLD alone. Findings confirm that poor phonology is the major cognitive risk factor for dyslexia.

Dyslexia is a neurodevelopmental disorder characterized by difficulties in learning to read and spell, typically associated with phonological deficits (Snowling & Hulme, 2012; Vellutino, Fletcher, Snowling, & Scanlon, 2004). However, although a phonological deficit appears to be the major proximal causal risk factor for dyslexia, there is increasing evidence that dyslexia may be the product of multiple risk factors, and children with a broader range of cognitive and sensorimotor deficits are more likely to develop reading problems (e.g., Carroll, Solity, & Shapiro, 2016; Pennington, 2006; Pennington et al., 2012; Snowling, 2008; van der Leij et al., 2013).

Important evidence regarding the development of reading difficulties comes from studies of children at family risk (FR) of dyslexia. These studies follow preschool children at high risk of developing reading disorder, comparing them to children at low risk, starting before they learn to read. Such studies are less subject to recruitment bias than case–control studies and therefore provide critical evidence regarding the skills that are the foundation of literacy before learning to read exerts a reciprocal influence on these cognitive abilities.

Snowling and Melby‐Lervåg (2016) reviewed 15 studies of children at FR of dyslexia that had followed children from preschool through Grade 1 or later. In addition to the widely reported phonological deficits, a meta‐analysis showed that children who go on to be classified as having dyslexia experience a broad range of language difficulties. There is also suggestive evidence from neurophysiological studies that biomarkers of dyslexia evident in infancy include difficulties in processing speech sounds (Leppänen et al., 2011; van der Leij et al., 2013 for reviews). Together, these findings are consistent with the idea that dyslexia is a form of language learning disorder.

Bishop and Snowling (2004) proposed that two sets of skills (two‐dimensions [2‐D]) have to be taken into account to explain the relationship between dyslexia and language disorder (SLI, now referred to as Developmental Language Disorder [DLD]; Bishop, Snowling, Thompson, Greenhalgh, & CATALISE‐2 Consortium, 2017). At the core of learning to read are phonological skills, whereas broader oral language skills (semantics and grammar) are required for reading comprehension. Children with dyslexia are like children with DLD in having poor phonological skills, consistent with the core phonological deficit hypothesis of Stanovich and colleagues (e.g., Stanovich, 1994; Stanovich, Siegel, & Gottardo, 1997). However, in “classic” dyslexia, language skills are unimpaired (as is reading comprehension except insofar as it is limited by decoding). In contrast, both of these skills are weak in DLD, such that these children experience a double deficit, leading to both impaired decoding and reading comprehension. A third type of reading disorder that specifically affects reading comprehension occurs when phonological skills are strong but language skills are impaired (the “poor comprehender” profile, Nation, 2005). Thus, within the “two‐dimensional model,” dyslexia and DLD are characterized by shared phonological deficits with broader language deficits being an additional characteristic of DLD.

Using data from a longitudinal population study, Catts, Adlof, Hogan, and Weismer (2005), compared the Bishop and Snowling (2004) 2‐D model with (a) the “severity” model that proposes that dyslexia and DLD are both caused by phonological deficits, DLD being the outcome when phonological difficulties are more severe, and (b) the “comorbidity” model suggesting that dyslexia and DLD can co‐occur and hence also dissociate (e.g., Bishop, McDonald, Bird, & Hayiou‐Thomas, 2009; McArthur, Hogben, Edwards, Heath, & Mengler, 2000; Ramus, Marshall, Rosen, & van der Lely, 2013). Following assessments in Grade 2 of children diagnosed in kindergarten with language difficulties and controls, children were classified into four groups: dyslexia, DLD‐normal reader, DLD + dyslexia, and normal reader. Although there was a raised incidence of dyslexia in the DLD groups (about 17% compared with a base rate of 8.6%), many children with DLD did not reach criteria for dyslexia. Given the limited overlap, Catts et al. (2005) suggested the severity hypothesis should be rejected. Turning to a comparison of the subgroups, the DLD‐normal reader group showed poor performance relative to controls in phoneme awareness (PA) in kindergarten and in nonword repetition in Grade 2, but these deficits were always less severe than those of the dyslexia groups and appeared to normalize over time. At no time point did the DLD‐normal reader group differ in language skills from the DLD + dyslexia group. The authors conclude that the data are in line with Bishop and Snowling's (2004) model in that dyslexia and language disorder share phonological deficits but favor the comorbidity model in that DLD is not necessarily associated with poor phonology (or dyslexia).

The findings of Catts et al. (2005) were replicated and extended by Bishop et al. (2009) who compared the language profiles of 9‐ to 10‐year olds identified as having dyslexia to those of children with DLD (with and without dyslexia) and typically developing (TD) readers of the same age. There were no significant differences between the children with dyslexia and those with DLD + dyslexia on reading and related measures (except reading comprehension). Similarly, there were few significant differences between the two language‐disordered groups on language measures; however, the DLD without dyslexia (DLD‐only) group showed better performance on tests of oromotor skill and repeating sentences as well as in performance in rapid naming (RAN), a skill known to predict learning to read; indeed, on this task, the children with DLD‐only performed like TD controls. Furthermore, their word‐level decoding skills were unimpaired.

Data were reported by Bishop et al. (2009) for the DLD and TD groups at ages 4 and 6 from the TEDS twin study (e.g., Viding et al., 2004). Looking back at the performance of the two DLD groups, they did not differ on any language measure at age 4 nor did they differ on nonword repetition or phonological awareness at age 6. However, the group later classified as having dyslexia was again poorer in oromotor skills. The authors concluded that poor language need not hinder the development of word‐level reading provided that RAN skills are normal.

In a similar vein, Ramus et al. (2013) made a direct comparison between 9‐ to 10‐year‐old children with dyslexia, children with DLD and children with comorbid dyslexia + DLD on tasks tapping phonological and broader oral language skills (including vocabulary, grammar, and syntax). Consistent with the aforementioned studies, children with dyslexia (with or without DLD) had poor phonological skills and children with DLD (with or without dyslexia) had poor broader language skills. Like Bishop et al. (2009), the authors report differences between the profiles of children with dyslexia and children with DLD, leading them to suggest that the nature of the phonological deficit differs in dyslexia and in DLD. Further evidence comparing the developmental trajectories that lead to dyslexia and language disorder is needed to ascertain more precisely the differences between these two disorders.

The different hypotheses about the relationship between dyslexia and DLD are not necessarily mutually exclusive; given the heterogeneity of both dyslexia and DLD, it could be that dyslexia is the outcome for children with more severe yet specific phonological difficulties, whereas a similar outcome could ensue when a milder phonological deficit is exacerbated by poor language. Furthermore, the possibility that risk factors outside of the language domain are relevant to predicting which children succumb to reading and/or language problems needs to be tested. For example, within the multiple‐risk framework (e.g., Pennington & Bishop, 2009), it may be that children with poorer attention or executive skills have fewer resources to bring to the task of learning to read and hence are more likely to experience failure.

In this article, we use data from a longitudinal study of children at high risk of dyslexia from age 3½ to 8 years to trace the development of word‐level reading problems from an early stage in development. We defined “high risk” to include children at FR of poor reading and children with preschool language concerns (who were therefore at risk of developing DLD as well as of having reading difficulties). Snowling and Melby‐Lervåg (2016) highlighted several methodological issues that studies of children at risk of reading difficulties should take into account. These include screening for co‐occurring conditions and avoiding the use of single measures that may be of low reliability. With these issues in mind, and given the language difficulties associated with dyslexia, our particular concern is the differentiation of dyslexia outcomes from those of DLD. First, we took steps to recruit families from a wide social spectrum, and we assessed all consenting parents for language and literacy problems. Second, we took the further precaution of assessing all children referred to the study for comorbid language difficulties (regardless of referral route) and collected data throughout the study on co‐occurring problems of attention and motor skills. Finally, we used multiple measures of each construct in order to use factor scores with increased reliability for investigating differences between children with different outcomes.

Our primary interest was to investigate shared risk factors for poor reading (decoding) between dyslexia and DLD. Accordingly, we classified “at‐risk” children recruited in preschool into those who at 8 years fulfilled criteria for dyslexia, DLD, dyslexia + DLD, or typical reader. We then analyzed differences at earlier phases of development between each disorder group and a control group of typical readers with neither a family history of dyslexia nor a history of developmental language problems.

Taking the findings of previous FR studies and studies comparing dyslexia and DLD as a starting point, we addressed the following research questions:

1. What is the incidence of dyslexia, defined as a word‐level reading deficit in children at FR, children with preschool language difficulties, and controls?
2. What is the rate of comorbidity between dyslexia and DLD?
3. Do the outcome groups differ in terms of a history of co‐occurring deficits in executive skills and attention or motor skills?

In addition, we specify the following hypotheses:

1. Poor phonology is a risk factor shared between dyslexia and DLD. That is, both children with DLD and those with dyslexia will show weaker performance on phonological skills than controls; children with both DLD and dyslexia will have the weakest phonological skills.
2. RAN deficits are specifically associated with dyslexia: Children with DLD alone will show stronger performance on RAN than either of the dyslexic groups.
3. Dyslexia is more likely to be diagnosed in children with language difficulties that persist at school entry, consistent with the critical age hypothesis (Bishop & Adams, 1990).

## Method

Data are reported from five phases of the Wellcome Language and Reading Project (*t*1–*t*5). Ethical clearance for the study was provided by the University of York, Department of Psychology's Ethics Committee and the NHS Research Ethics Committee. Parents provided informed consent for their child to be involved. Children were assessed by an experienced research team who were trained and observed by the project manager to ensure fidelity. Testing took place at approximately annual intervals: in the children's homes at *t*1 and *t*2 (at 3½ and 4½ years) and usually at school at *t*3–*t*5 (5½–8 years); where possible, the same assistant visited the child on each occasion. Each child was administered cognitive, language, and literacy tests at each time point (tests of perceptual processing and numeracy are the subject of other articles and are not discussed here). At *t*1, the assessment was administered in a single 90‐min session; at *t*2, across two 1‐hr sessions, with breaks; and at *t*3–*t*5 in a 2‐hr session, tests normally given in a fixed order.

### Participants

Families were recruited to the study between 2007 and 2009 from the City of York and neighboring regions within Yorkshire and the northeast of England. At the time, most families living in the area were monolingual and all children spoke English as their first language (the majority of the sample were White British). Recruitment was via advertisements placed in local newspapers, nurseries, the web pages of support agencies for children with reading and language difficulties, and via speech and language therapy services. Based on our previous study of children at FR of dyslexia, we expected large effect sizes for comparisons of dyslexia and control groups (*d *=* *1.18; single word reading). Sample sizes of 100 versus 75 children in the risk group were estimated to provide 90% power to detect a difference of 0.5 *SD* units between the FR and control groups (α* *=* *.05 two‐tailed). Sample sizes of 75 children in the control and language impaired groups provided 90% power to detect a difference of 0.54 *SD*s between the FR and control groups (α* *=* *.05 two‐tailed). A sample size of 250 was feasible within the time frame allotted to the recruitment phase.

Of the 260 children recruited, none met exclusionary criteria at *t*1 (MZ twinning, chronic illness, deafness, English as a second language, care provision by local authority, and known neurological disorders such as cerebral palsy, epilepsy, and autism spectrum disorder). The educational level of both parents was collected during a family interview, based on a scale ranging from 1 (*no formal qualifications*) to 6 (*postgraduate qualification*). Additionally, data on the best occupational status of both parents were collected, using the Standard Occupational Classification (Office for National Statistics, 2010). This scale ranges from 1 (*unemployed*) to 10 (*managers, directors, senior officials*). Best occupational status was preferred to current occupational status as an indicator of socioeconomic status (SES), because many of the parents interviewed were on parental leave from work at the time of data collection. The full range of SES was represented in the sample, although all variables showed a negative skew, indicating relatively high average SES.

**Figure 1:**
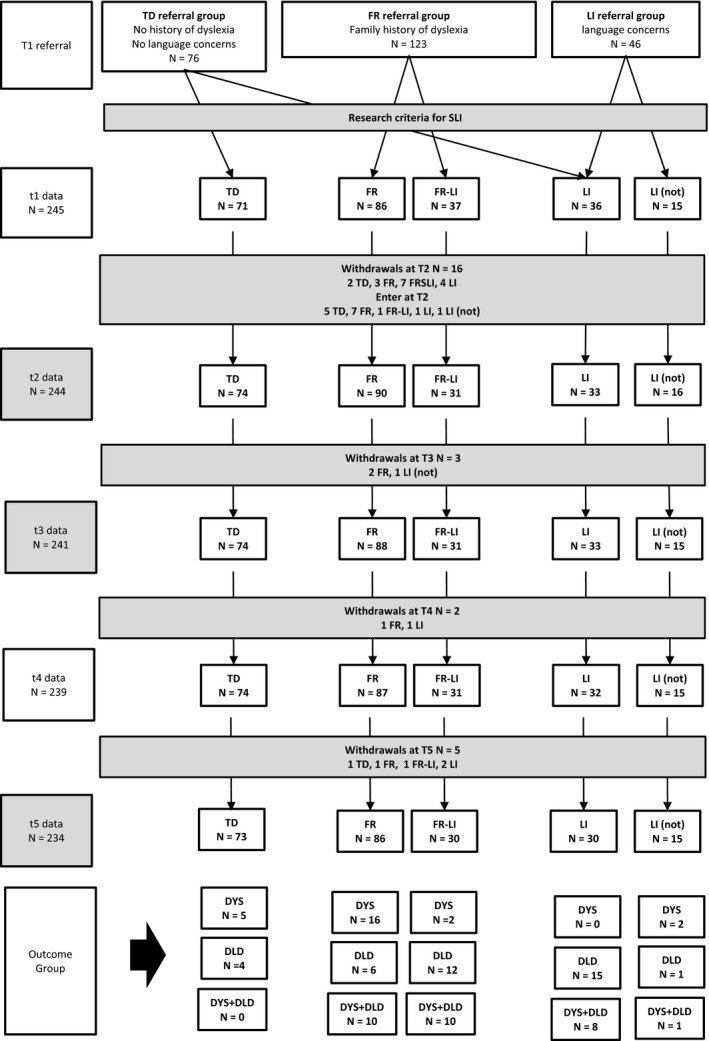
Participant flow diagram. TD = typically developing; FR = family‐risk; LI = language impairment; DLD = Developmental Language Disorder; DYS = Dyslexia.

Following recruitment, children were classified using a two‐stage process: first to determine whether they were at FR of dyslexia and then to ascertain whether they had a preschool language impairment (LI) placing them at risk of DLD. This led to the classification of children into four groups: FR‐only (*N *=* *86, 50M, 36F); FR‐LI (*N *=* *37, 26M, 12F); LI‐only (*N *=* *36, 26M, 10F), and TD (*N *=* *71, 35M, 369F); details of their preschool language profiles are described in Nash, Hulme, Gooch, and Snowling (2013). In addition, a small group of 15 children (9M, 6F) referred for language difficulties did not reach research criteria for diagnosis of a LI. There was a small amount of attrition between time points, which was greatest between *t*1 and *t*2 (*N* = 16) and reduced between later assessments (*t*2–*t*3 *N *=* *3, *t*3–*t*4 *N *=* *2, *t*4–*t*5 *N *=* *5). Data from all children recruited to the study who remained in the sample (including those who failed to reach diagnostic criteria at *t*1) are included in the present analyses (*N *=* *234; see Figure 1 for participant flow).

#### Family‐Risk

A child was classified as at FR if (a) a parent self‐reported as “dyslexic” on the Adult Reading Questionnaire (Snowling, Dawes, Nash, & Hulme, 2012), (b) a parent scored below 90 on a literacy composite comprising nonword reading and spelling, (c) a parent had a discrepancy between nonverbal ability and the literacy composite of 1.5 *SD*s, with a literacy composite standard score of 96 or below, or (d) a sibling had a diagnosis of dyslexia from an educational psychologist or a specialist teacher.

#### Risk of DLD

Children were classified as language impaired in preschool (at risk of DLD) if they scored below the cutoff on two of four of the following language tests: Clinical Evaluation of Language Fundamentals (CELF)–Preschool 2 UK, Basic Concepts, Expressive Vocabulary, Sentence Structure (Semel, Wiig, & Secord, 2006), and the screener from the Test of Early Grammatical Impairment (TEGI; Rice & Wexler, 2001). The cutoff for the three CELF subtests was a scaled score below 7; for the TEGI, a combined score across third person singular and past tense subtexts of 62%, according to the manual 80% sensitivity and specificity. (In a small number of cases where there was missing data, information from other language tasks was used.) Based on these criteria, 35/120 children at FR and 32/46 children recruited with language concerns reached criterion for LI. In addition, of the 76 children initially classified as TD, five met these criteria. These children were considered to be at risk of DLD and placed in the LI group (for further details, see Nash et al., 2013).

#### Classification of Outcomes at *t*5 (age 8)

Dyslexia status at *t*5 was defined by performance on the Single Word Reading Test (SWRT 6–16; Foster, 2007) and the Wechsler Individual Achievement Test, 2nd ed. (WIAT–II) Spelling Test (Wechsler, 2005). The average of the age‐standardized scores formed a composite literacy score (TD mean* *= 106.88, *SD *=* *11.68). Dyslexia was defined as falling at > 1.5 *SD* below the mean (a score of 89 or less). Of the 234 children remaining in the sample, 50 were identified as having dyslexia according to this criterion, and 184 were normal readers.

DLD at *t*5 was defined by performance on a composite language measure formed by averaging the age‐standardized scores from *Expressive Vocabulary* (*CELF–4, UK*—Wiig, Semel, & Secord, 2006), Test for Reception of Grammar, Version 2 (TROG–2—Bishop, 2003), and *Formulated Sentences* (*CELF–4*). DLD was defined as falling −1 *SD* below the mean of the composite language score (Snowling, Duff, Nash, & Hulme, 2016). Using this criterion, 67 children were classified as having DLD, and 167 as having normal language.

### Tests and Procedures

The tasks administered to assess each construct at each time point are shown in Table S1; where possible the same test was used at each time point but some alternative tests were introduced to avoid ceiling and practice effects. Brief details, together with test reliabilities follow (full details in the Appendix S1).

#### Nonverbal Ability

Each child completed tasks from the Wechsler Scales of Intelligence (Wechsler Preschool and Primary Scale of Intelligence, 3rd ed., Wechsler, 2002; Wechsler Intelligence Scale for Children, 4th ed., Wechsler, 2003). At *t*1, the Block Design and Object Assembly subtests were used (split‐half reliability Block Design = .84; Object Assembly = .85) and at *t*5, Block Design and Matrix Reasoning (split‐half reliability Block Design = .89; Matrix Reasoning = .89).

#### Language

##### Comprehension

At *t*1, *CELF Basic Concepts* (*CELF*–*Preschool 2 UK*; Wiig et al., 2006) was given. The child heard a sentence and had to select from a choice of three, the picture that represented the concept (α = .81–.87).

##### Receptive grammar

The child heard sentences of increasingly complex syntactic structure and had to select from a choice of four pictures the one that conveyed the meaning of each. The tests were: at *t*1–*t*2 *Sentence Structure* (*CELF*–*Preschool*, Wiig et al., 2004); at *t*3, *Sentence Structure* (*CELF–4*, Wiig et al., 2006; α = .83); and at *t*4–*t*5, the *TROG–2* (Bishop, 2003; α = .88).

##### Expressive grammar

The child repeated sentences of increasingly complex syntactic structure. At *t*1 and *t*2, the Sentence Imitation Test (SIT‐16; Seeff‐Gabriel, Chiat, & Roy, 2008; α = .92); at *t*3–*t*5, the experimental sentence imitation test, *ESIT*, a test designed for the study that requires repetition of 20 sentences: 10 (five long/five short) containing transitive verbs and 10 (five long/five short) containing ditransitive verbs (α = .78). The score was number of sentences repeated correctly.

##### Morphological inflection

At *t*1, *t*2, and *t*3, the ability to produce grammatical inflections (third person/past tense) was assessed using the *TEGI* (Rice & Wexler, 2001; split‐half *r *=* *.82 [past tense] and .92 [third person singular]). At *t*4, *CELF–4 Word Structure* (α = .78–.86); and at *t*5, *CELF–4 Formulated Sentences* (α = .76) measured expressive grammar.

##### Vocabulary

Tests of receptive and expressive vocabulary were given. At *t*1, *t*3, *t*4, and *t*5, the *CELF Expressive Vocabulary* test (*CELF*–*Preschool 2 UK*, Wiig et al., 2006; *CELF–4*, Semel et al., 2006; Wiig et al., 2006) at *t*3 (α = .84), with extension items at *t*4, *t*5 (α = .66); at *t*2, *t*4, and *t*5, the Receptive One Word Picture Vocabulary Test (Brownell, 2000; α = .95).

#### Phonology

##### Speech

At *t*1, the articulation subtest of the Diagnostic Evaluation of Articulation and Phonology (*DEAP*; Dodd, Hua, Crosbie, Holm, & Ozanne, 2002) requiring the child to name or imitate the names of 30 pictures was given; percentage of consonants correctly produced was recorded. At *t*2, the screening test from the *DEAP* was administered and further assessment was undertaken of any child with current speech problems (most articulatory errors are resolved by this age).

##### Repetition

At *t*1 and *t*2, the child repeated 18 words and 18 nonwords from the *Early Repetition Battery* (Seeff‐Gabriel et al., 2008; α = .89), and at *t*3, the nonwords only. Consistent articulation errors were taken into account when scoring the children's responses. At *t*3–*t*5, children repeated nonwords (3–5 syllables in length) from the new nonword repetition test, *NNWRep*, a task devised for this study (αs = .77–.80) and at *t*5, the Children's Nonword Repetition Test (Gathercole & Baddeley, 1990) was also given (test–retest *r *=* *.77; split‐half *r *=* *.66).

Our measures of Phonology index various aspects of a child's ability to process and produce the phonological structures of English. These differ from the following measures of Phonological Awareness, which additionally draw on metacognitive skills.

#### Phonological Awareness

At *t*2, the child completed syllable and alliteration matching tasks to assess emergent phonological awareness (Carroll & Snowling, 2001). At *t*2 and *t*3, they completed a Phoneme Isolation task, in which the child was asked to repeat a nonword and then say its first (initial phoneme) or last (final phoneme) sound (α = .71–.93). At *t*3–*t*5, they completed the phoneme deletion task from the *York Assessment of Reading for Comprehension* (*YARC Early Reading*; Hulme et al., 2009; α = .95) with extension items at *t*4–*t*5 to avoid ceiling effects.

#### Rapid Automatized Naming

At *t*2, there were two versions of this task: colors and objects. Children named an 8 × 5 array of 40 stimuli as quickly as possible for two trials each. RAN rate was calculated as the mean number per second. RAN objects were given at *t*3, *t*4, and *t*5; at *t*4 and *t*5, the children also completed a RAN digit task.

#### Literacy

##### Letter knowledge

At *t*1, the child was asked to give the sound of 12 letters (α = .95). At *t*2, *t*3, and *t*4, they gave the sound of 32 single letters and digraphs (*YARC*; Hulme et al., 2009; α = .92).

##### Word‐level reading (decoding) skills

At *t*2, *t*3, and *t*4, the child completed the *YARC* Early Word Reading (Hulme et al., 2009) comprising 15 regular and 15 irregular words; at *t*3, *t*4, and *t*5 (α = .98), the S*WRT* (Foster, 2007) comprising 60 words (α = .98) and at *t*5, the Exception Words from the *Diagnostic Test of Word Reading Processes* (Forum for Research into Language and Literacy, 2012) was also given (α = .97).

##### Spelling

At *t*3, the child was asked to spell five words (*dog*,* cup*,* tent*,* book*,* heart*), each represented by a picture (α = .53); at *t*4, 12 words (α = .86). At *t*5, the WIAT of Spelling was given (Wechsler, 2005; α = .96).

#### Motor Skills

##### Fine motor skill and eye–hand coordination

At *t*2, *t*3, and *t*4, children's hand preference was recorded and three subtests from the Movement Assessment Battery for Children, 2nd ed. (ABC–2; Henderson, Sugden, & Barnett, 2007) were included to assess fine motor skill and eye–hand coordination: (a) *Posting Coins*, (b) *Bead Threading,* and (c) *Bicycle Trails* using their preferred hand to draw a single continuous line, following a trail without crossing its boundaries. Errors were recorded according to scoring guidelines.

#### Executive Function

##### Selective attention

At *t*1–*t*4, the child was assessed using a visual search task (the *Apples Task*; Breckenridge, 2008). A visual search efficiency score ((Hits: total targets correctly identified − commission errors)/60 s) was calculated (stability; *r *=* *.59).

##### Sustained attention

At *t*2, *t*3, and *t*4, the child completed an Auditory Continuous Performance Test (ACPT) devised for this study. Children saw an image of a farm and heard four different animal sounds; they pressed a button on a button box when they heard a dog bark. A sustained attention efficiency score ((Hits: total targets correctly identified (max 30) − commission errors)/120 trials) was calculated.

##### Behavioral inhibition/self‐regulation

At *t*1, the child was assessed on the *dog/bird* task. The child had to inhibit their natural inclination to follow verbal instructions for motor/hand actions (e.g., “thumbs up”) given by one puppet (a bird) while responding to commands given by another (a dog). An efficiency score (Hits: number of responses to the dog (max 8)/total number of responses (responses to dog + bird (max 16)) is calculated. At *t*4 and *t*5, the child completed a *Go/No‐Go* task in which they had to press a button as quickly as possible (within 2,000 ms) when they saw a “bug” but not when they saw a “ladybird.” The number of commission errors made on No‐Go trials was used as an index of behavioral inhibition.

At *t*1, *t*2, and *t*3, the child completed the Head, Toes, Knees, Shoulders task (Burrage et al., 2008), a measure of behavioral regulation. The child heard verbal commands and was instructed to do the opposite action (e.g., touch their toes if asked to touch their head). Children scored two points for a correct response and one point for a self‐corrected response (max score* *=* *40; stability: *r *=* *.52).

##### Visual‐spatial memory

At *t*2–*t*5, the *Block Recall* task from the Working Memory Test Battery for Children (Pickering & Gathercole, 2001) was given (α = .63). The child saw the examiner tap a sequence of blocks on a board and then recalled the sequence by tapping the blocks in the same order. The number of correct trials was recorded (max 52).

### Factor Scores

Factor analysis (principal factor method, without rotation) was used to derive a factor score for each of the seven separate domains (see Table 1). The factor for each domain captures the common variance that is shared between all measures.

**Table 1:** Measures Used to Derive Factor Scores for Analyses

Factors	T1	T2	T3	T4	T5
Language	Expressive Vocabulary Sentence Structure (CELF–P‐2) TEGI	Receptive Vocabulary (ROWPVT) Sentence Structure (CELF–P‐2) TEGI	Expressive Vocabulary Sentence Structure (CELF–4) TEGI	Expressive Vocabulary Word Structure (CELF–4) TROG–2	Expressive Vocabulary Formulated Sentences (CELF–4) TROG–2
Phonology	Percent consonants correct (DEAP) Word and nonword repetition (PSRep)	Percent consonants correct (DEAP) Word and nonword repetition (PSRep)	Nonword repetition (PSRep and NNWRep)	Nonword repetition (NNWRep)	Nonword repetition (NNWRep and CNRep)
Phonological awareness	—	Alliteration matching Phoneme isolation	Phoneme isolation Phoneme deletion (YARC)	Phoneme deletion (YARC + extension items)	Phoneme deletion (YARC+extension items)
Rapid automatized naming (RAN)	—	RAN objects RAN color	RAN objects (Trial 1, Trial 2)	RAN objects RAN digits	RAN objects RAN digits
Decoding	—	Regular words (EWR) Irregular words (EWR)	Regular words (EWR and SWRT) Irregular words (EWR)	Word reading total (EWR and SWRT)	Word reading (SWRT) Exception word reading
Motor skills	Posting coins Threading beads Bicycle trail (ABC–2)	Posting coins Threading beads Bicycle trail (ABC–2)	Posting coins Threading beads (ABC–2)	Posting coins Threading beads (ABC–2)	—
Executive function	Selective attention (visual search efficiency) Inhibition (dog/bird task efficiency) Self‐regulation (HTKS)	Selective attention (visual search) Sustained attention (ACPT) Self‐regulation (HTKS) Visuo‐spatial memory (block recall)	Selective attention (visual search) Sustained attention (ACPT efficiency) Self‐regulation (HTKS) Visuo‐spatial memory (block recall)	Selective attention (visual search) Inhibition (Go/No‐Go commission errors) Visuo‐spatial memory (block recall)	Selective attention (visual search) Inhibition (Go/No‐Go commission errors) Visuo‐spatial memory (block recall)

Note

ROWPVT = Receptive One Word Picture Vocabulary Test; CELF–4 = Clinical Evaluation of Language Fundamental, 4th ed.; CELF–P‐2 = CELF Preschool 2; TEGI = Test of Early Grammatical Impairment; EWR = Early Word Reading; TROG–2 = Test for Reception of Grammar, Version 2; DEAP = Diagnostic Evaluation of Articulation and Phonology; YARC = York Assessment of Reading for Comprehension; SWRT = Single Word Reading Test; ABC–2 = Assessment Battery for Children, 2nd ed.; HTKS = Head, Toes, Knees, Shoulders; ACPT = Auditory Continuous Performance Test; CNRep = Children's Nonword Repetition.

## Results

We begin by presenting data on the incidence of dyslexia and DLD in the current sample and then compare the performance of the children with dyslexia, DLD, and comorbid dyslexia + DLD with controls at each of the assessment points (*t*1–*t*5). Analyses are conducted on factor scores (using the principal factor method) derived from the whole sample except where otherwise stated.

We first report the outcomes of the children in the study recruited to be at high risk of dyslexia compared with controls and discuss the associated risk factors. We then present the findings of between‐group analyses assessing language, literacy, executive, and motor skills at the five points of the study. For reasons of space, we report only the effect size of the deficit for each group on each factor at each time point. The full data set and results of between‐group analyses are given in the Appendix S1.

### Language and Literacy Outcomes of Children at High Risk of Dyslexia

Table 2 (upper rows) shows the performance of children from the TD and risk groups, as defined in preschool, on key outcome measures of language and literacy at *t*5, together with data from the small group who were referred because of speech‐language problems but did not fit research criteria for either FR or LI.

**Table 2:** Outcomes at *t*5 According to Risk Group at *t*1 (3½ Years)

	Classification at *t*1				Referred as LI but did not reach criterion^3^ (*N* = 15)	ANOVA *F*(3,215)^4^
	TD (*N* = 73)	FR (*N* = 86)	LI (*N* = 30)	FR‐LI (*N* = 30)		
*t*5 Measures						
Language^1^	0.50 (0.61)_1_	0.17 (0.77)_2_	−0.84 (0.84)_3_	−0.91 (0.74)_3_	0.07 (0.83)	42.11
Phoneme awareness^1^	0.44 (0.67)_1_	−0.09 (0.85)_2_	−0.46 (0.90)_3_	−0.33 (0.69)_3_	−0.08 (0.85)	13.38
Decoding^1^	0.43 (0.66)_1_	−0.04 (1.04)_2_	−0.23 (1.02)_2,3_	−0.55 (0.92)_3_	−0.19 (1.04)	9.31
Spelling^2^	103.71 (13.13)_1_	96.96 (13.85)_2_	93.48 (16.66)_2,3_	90.23 (11.88)_3_	95.53 (17.92)	8.58
Performance IQ^2^	110.02_1_ (12.79)	104.12_2_ (13.90)	92.6 (14.84)_3_	95.0 (14.73)_3_	107 (13.40)	15.54
Outcome groups at *t*5						
Dyslexia (total *N*)	5 (6.8%)	22 (25.6%)	8 (26.7%)	12 (40%)	3 (20%)	—
Dyslexia‐only (*N*)	5 (6.8%)	12 (13.9%)	0	2 (6.7%)	2 (13.3%)	—
DLD (total *N*)	5 (6.8%)	16 (18.6%)	23 (76.7%)	22 (73%)	2 (6.7%)	—
DLD‐only (*N*)	5 (6.8%)	6 (7.0%)	15 (50%)	12 (40%)	1 (6.7%)	—
Dyslexia + DLD	0	10 (11.6%)	8 (26.7%)	10 (33.3%)	1 (6.7%)	—

Note

TD = typically developing; FR = family‐risk; LI = language impairment; DLD = Developmental Language Disorder; ANOVA = analysis of variance.

^1^Factor (*z*) score ^2^Standard score ^3^Excluded analyses at *t*1 ^4^ANOVA, excluding children with speech‐language concerns; values with same subscript do not differ significantly.

As expected, the TD control group, who were recruited for being at low risk of reading disorder, performs better on measures of PA, decoding, and spelling than children in the risk groups and has stronger language skills. Although they are of higher nonverbal IQ than the language impaired groups, they do not differ statistically significantly from the FR‐only group in nonverbal IQ or social background. Generally, the FR‐only group performs better than the DLD risk groups in language, PA, and decoding and is of higher nonverbal ability. However, differences from the preschool LI group in decoding and spelling are not significant; it is also of interest to note that the group of children who were referred for speech‐language concerns performs similarly to the FR‐only group at this age—less well than controls. Finally, the FR‐LI group performs least well across all tasks but not significantly differently from the LI‐only group. In short, the general pattern across language and literacy is for the TD group to perform best, followed by the FR group, with the two preschool LI groups being most impaired.

The bottom rows of Table 1 show the total numbers of children in the TD and each of the risk groups classified as having dyslexia or DLD at *t*5 for comparison with earlier studies. We also show the diagnostic groups broken down into pure and comorbid conditions.

In the TD control group, that is, children who did not fulfill criteria either for FR or for preschool LI, 6.8% in this sample fulfill criteria for dyslexia. Summing across FR subgroups (FR and FR‐LI), the incidence of dyslexia is 29% for children at familial risk of dyslexia and 33% for children with preschool LI. Turning to risk of DLD: 7% of the TD group were affected and 19% of the FR group who did not reach criteria for preschool LI were now identified as having DLD. Summing across the preschool LI groups (LI and FR‐LI), 75% reach criteria for DLD. However, a substantial number of children have comorbid dyslexia with DLD at *t*5; the bottom three rows for the table show the numbers in each risk group who develop pure versus comorbid disorders.

### Classification of Outcomes: Dyslexia and DLD

Table 3 shows the numbers of children in the whole sample (including the group of children with speech‐language concerns at *t*1 who did not fulfill research criteria for LI) classified as having dyslexia and/or DLD at *t*5).

**Table 3:** Categorical Classification of Dyslexia Versus Developmental Language Disorder (DLD) Showing Comorbidity at *t*5, With Gender Ratios

	No DLD	DLD	Total
No dyslexia	146 (77M:69F)	38 (23M:15F)	184
Dyslexia	21 (14M:7F)	29 (22M:7F)	50
Total	167	67	234

Of the 50 children who met criteria for dyslexia (as defined by the word‐level measure of reading and spelling), 21 had TD language skills (dyslexia‐only) and 29 met criteria for comorbid DLD (dyslexia + DLD). A further 38 children met criteria for DLD but did not have dyslexia (DLD only). The gender ratios for each subgroup are also shown in the table. Approximately equal numbers of boys and girls are classified as normal readers; for dyslexia, the ratio is 2:1 boys to girls affected if language is unimpaired and ~3:1 for those with DLD. There was an overall group difference in SES (Table S2); the group with dyslexia did not differ in SES from TD controls although there was a trend in this direction, whereas both DLD groups were of lower SES than TD controls. The difference in SES between the group with dyslexia and the DLD and comorbid groups was not statistically significant.

Data were available on the language trajectories of 220 of the sample from *t*1 through *t*3 to *t*5 (see Snowling, Duff, Nash & Hulme, 2016 for details). Some children with language difficulties at *t*1 have resolved those difficulties by *t*5 (resolving trajectory), whereas other children have persisting DLD (persisting trajectory). Of the 12 children with a resolving language trajectory, only one met criteria for dyslexia at *t*5, whereas 17 of the 42 with persisting language difficulties did so (40%).

### Developmental Profiles of Dyslexia and DLD

Retrospective analyses focusing on preschool precursors (*t*1, *t*2) and school‐age differences (*t*3, *t*4) between children classified with dyslexia and/or DLD at *t*5 are presented next. In these analyses, as in most previous FR studies, we took as the “normal reader” comparison group, TD controls from *t*1 (because they were recruited as at low risk of dyslexia and DLD), excluding any child with dyslexia or DLD at outcome. In the sections that follow, data are presented graphically showing the effect size of the deficits in performance of the three outcome groups relative to this TD control group (the complete data set is provided in the Appendix S1). These tables include the performance of the children who are typical readers at *t*5, including those who were initially at risk.

#### Deficits in Language and Reading‐Related Skills for Children With Dyslexia and DLD

**Figure 2:**
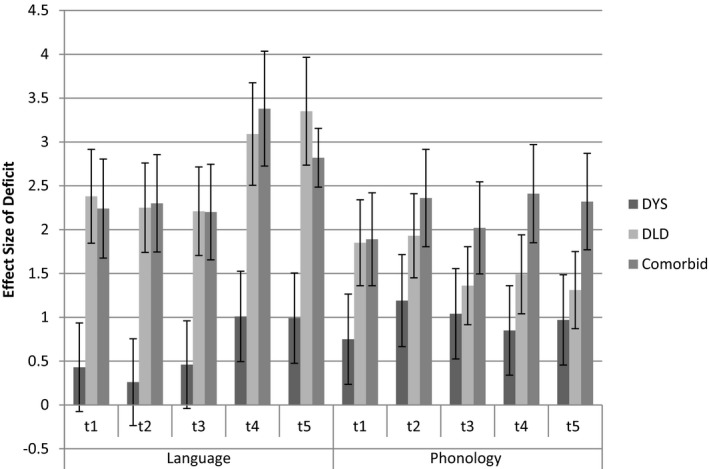
Effect size (with 95% confidence intervals) of group deficits in language and phonology measures at *t*1–*t*5 for dyslexia, Developmental Language Disorder (DLD), and comorbid groups.

Figure 2 shows the difference between the TD control group and the three outcome groups on language and phonological language tasks between *t*1 and *t*5 (Table S2 for group means and *SD*s). On measures of both language and phonology, the group with dyslexia‐only shows medium to large deficits relative to the TD controls, whereas the DLD‐only group shows large deficits on phonology and very large deficits on language; the dyslexia + DLD group shows very large deficits in both language and phonology (*d*s ranging from 1.8 to over 3).

For the group with dyslexia, language deficits are small in preschool, appear to increase with age, and are large after school entry; the DLD‐only and the dyslexia + DLD group started with poorer language and difficulties also increased over time. For phonology, the group with dyslexia‐only shows large deficits from preschool onwards, but the deficit is somewhat variable over time (*d*s = 0.75–1.09); the dyslexia + DLD group shows larger deficits (*d*s = 2.02–2.36). Although the DLD‐only group also shows large phonological deficits relative to TD controls, the deficit appears to lessen over time (*d*s = 1.93–> 1.3).

**Figure 3:**
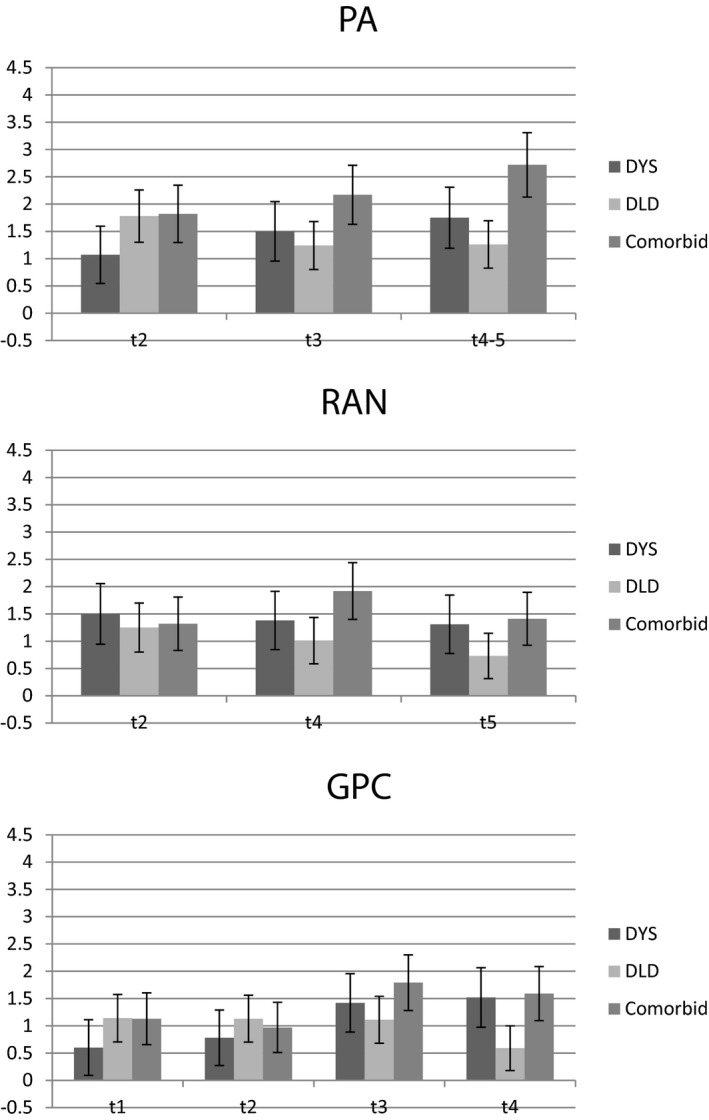
Effect size (with 95% confidence intervals) of group deficits in letter knowledge (upper panel), phonological awareness (middle panel), and rapid naming (lower panel) at *t*2–*t*5 for dyslexia, Developmental Language Disorder (DLD), and comorbid groups.

Figure 3 shows the effect sizes of differences between the TD control group and the three outcome groups in PA, rapid naming (RAN), and letter knowledge (GPC) over the course of the study. In preschool, all three clinical groups show large deficits in these skills and differences between the performance of the two groups with DLD and the dyslexia‐only group are not statistically significant. However, from *t*3 (age 5½ years) onwards the DLD‐only group tends to show smaller deficits on these foundation skills than either of the two groups with dyslexia.

**Figure 4:**
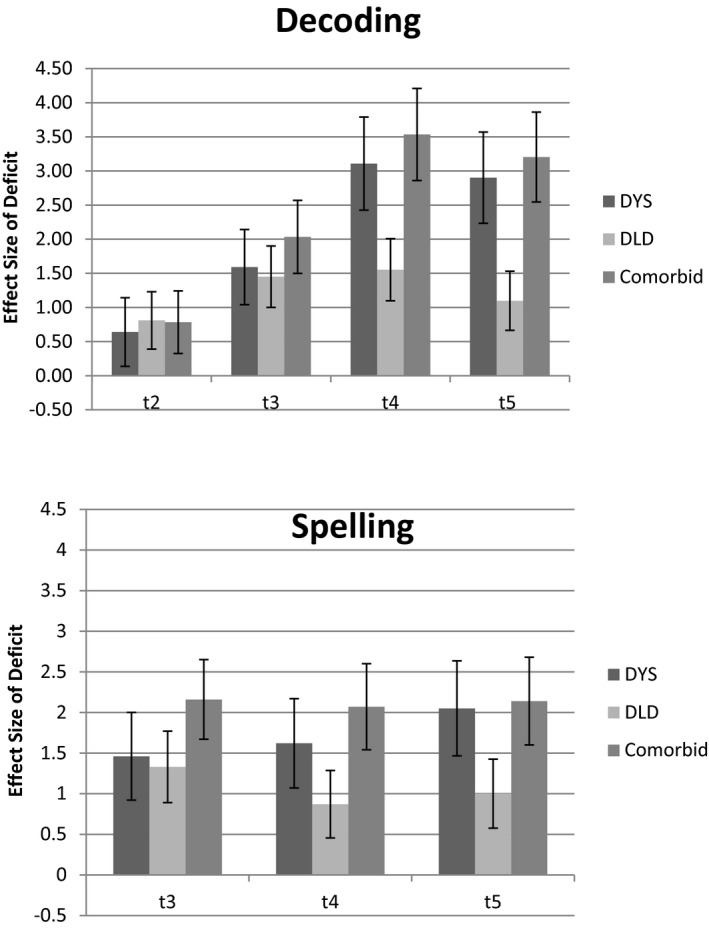
Effect size (with 95% confidence intervals) of group deficits in decoding (upper panel) and spelling (lower panel) skills at *t*1–*t*5 for dyslexia, Developmental Language Disorder (DLD), and comorbid groups.

In summary, children with dyslexia show phonological deficits from early in preschool before reading instruction begins. This is also true of the DLD‐only group although there is a trend for these to decrease over time for this group. Indeed, the relative improvements shown by the DLD‐only group in PA, GPC, and RAN contrasts with the pattern seen in the two groups with dyslexia in which these presage differences in the development of reading. Figure 4 shows the effect size of the deficits in decoding skills and spelling (observed measure) for the three groups over the same time period. Although the DLD‐only group have, by definition, better reading skills than the groups with dyslexia, they still show substantial deficits relative to TD controls in decoding and spelling. The dyslexia‐only and dyslexia + DLD groups have similarly severe deficits in literacy.

**Figure 5:**
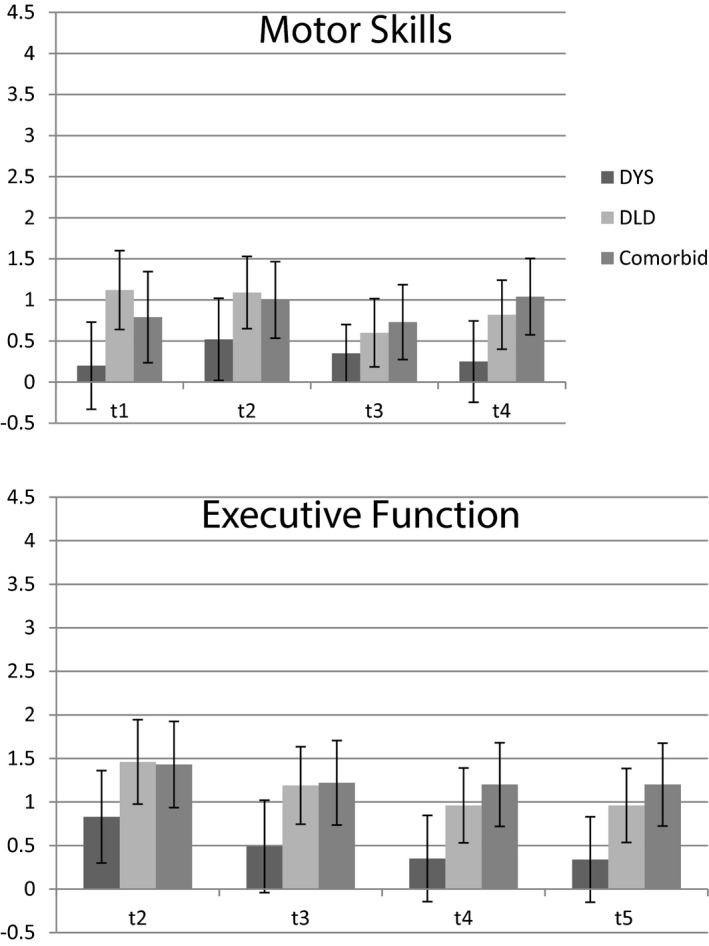
Effect size (with 95% confidence intervals) of the group deficits in motor (upper panel) and executive (lower panel) skills.

Figure 5 shows effect sizes for the deficits on the motor and executive tasks from *t*1 to *t*5. The performance of the group with dyslexia‐only is not statistically significantly different from that of TD controls (with the exception of executive skills at *t*2), although it is clear from the medium effect sizes that not all children with dyslexia are free of these impairments. In contrast, both groups with DLD show large deficits in these nonverbal skills at all assessment points.

## Discussion

In this study, we followed children at FR of dyslexia and children with preschool language difficulties from age 3½ to 8 years, comparing them with TD controls selected to be at low risk of reading and language disorders. We assessed the children twice before they entered school and three times during the school years. Children were classified according to both reading and language status when most were in the third year of formal schooling. Children with a poor language outcome (DLD), with or without dyslexia, show a wide range of impairments in the preschool period, including poor prereading skills and problems with executive and motor tasks outside of the verbal domain. The difficulties of children with a dyslexic outcome (without DLD) are more specific, although they show increasing difficulty on language tasks in the school years, and it is clear that some experience deficits beyond the verbal domain in motor and executive skills. For children with a dyslexic outcome, deficits in phonology, letter knowledge, PA, and RAN are relatively large even in the preschool period, and they show an increasing deficit in decoding over time, in contrast to the pattern observed for children with DLD who do not develop dyslexia.

Consistent with previous studies, we found an increased rate of reading difficulties among children at familial risk of dyslexia and also among children with a history of preschool LI (Pennington & Bishop, 2009). Among controls with neither risk, the incidence of dyslexia was 6.8%, whereas among those at familial risk it was 29%. These rates are lower than in previous FR studies in which a mean incidence of 11.6% for controls and 45% (range = 29%–65%) for children at FR is reported (Snowling & Melby‐Lervåg, 2016). One possible reason for the difference is that here we considered families to be “at‐risk” not only when parents self‐reported as having dyslexia but also when they also achieved poor scores on literacy measures—Snowling and Melby‐Lervåg (2016) also reported a lower rate for studies in which dyslexia was determined by formal assessment. Another possible reason is that many previous FR studies have small sample sizes such that one or two more cases could affect incidence rates; it is notable that the largest previous study reported an incidence rate of 35%, closer to the current estimate (Torppa, Lyytinen, Erskine, Eklund, & Lyytinen, 2010).

The incidence of dyslexia in children with preschool LI in our sample was 33%, which is closely comparable to the figures reported from the Iowa longitudinal cohort (Catts et al., 2005). Further, consistent with the high level of concurrent language difficulties reported in children referred for reading difficulties (McArthur et al., 2000), we found that 43% of children with DLD at age 8 also met criteria for dyslexia and 58% of children with dyslexia had DLD. Of the children with dyslexia, some 76% had significant language difficulties at 5½ years shortly after entering school, in line with the “critical age hypothesis” (Bishop & Adams, 1990). We also found increased vulnerability for both dyslexia and DLD in boys (Arnett et al., 2017; Rutter et al., 2004), and it is notable that children with DLD (regardless of dyslexia status) are of lower SES than children with dyslexia and typical readers (though their difference from the dyslexia‐only group is not statistically significant).

Together our findings confirm that poor language is a precursor of dyslexia, as originally proposed by Scarborough (1990). Further we show that dyslexia and DLD have shared deficits in phonological processing placing them at risk of poor reading. These findings are in line with a longitudinal model of reading development proposed by Hulme, Nash, Gooch, Lervag, and Snowling (2015) which shows that early measures of language and phonology at age 3½ predict decoding skills at age 5½ years and their impact is mediated by PA and grapheme‐phoneme skills.

Notwithstanding the association between poor language and poor reading, like Catts et al. (2005), we found that dyslexia does not always follow preschool LI. Although we show that difficulties in the early stages of learning to read are common for children with DLD, a novel finding is that dyslexia and DLD show different developmental trajectories. In dyslexia, a specific deficit in phonological aspects of language (e.g., in nonword repetition) is observed in the preschool period prior to reading instruction without problems of similar magnitude in the broader language domain (except where there is comorbidity with DLD). By contrast, in DLD, poor phonology in the preschool years is accompanied by very poor language (encompassing deficits in vocabulary and grammar). In DLD‐only, in contrast with dyslexia and the comorbid form, phonological difficulties decrease over time, though they are significant in comparison with TD children in the early years, as are problems in PA, letter knowledge, and RAN. Nonetheless, it is important to emphasize that the decoding difficulties observed in DLD only were always less severe than in dyslexia, arguably as a result of better PA and RAN. Later in development, we expect that reading comprehension will pose a particular challenge for these children despite their ability to decode because of underlying language difficulties; Catts, Fey, Tomblin, and Zhand (2002) reported that 48% of their sample of children with language difficulties in kindergarten had reading comprehension problems.

Turning to cases of comorbid dyslexia and DLD, we provide evidence that phonological deficits are more severe than in either condition alone. Decoding and spelling skills are impaired to the same extent as for the dyslexia group and more impaired than for the DLD‐only group, but their language skills are similar to those of the DLD‐only group and more impaired than in the dyslexia group. In terms of developmental profile, our findings confirm those of Bishop et al. (2009) who found no significant differences between the groups with DLD‐only and dyslexia + DLD in phonology in preschool. However, from age 5½, the DLD‐only group outperformed the comorbid dyslexia + DLD group in nonword repetition and PA, whereas this was not the case in Bishop's sample. Similarly, our findings reveal a trend for the DLD‐only group to do better on tests of rapid naming similar to previous studies. Although group differences did not reach statistical significance, the profile of phonological deficits appears to differ between dyslexia and DLD, as reported by Ramus et al. (2013). This is seen most clearly at *t*5 when the DLD‐only group has resolved their phonological problems to a large degree.

Regarding literacy outcomes, the pattern of differences between dyslexia and DLD is broadly in line with those of previous cross‐sectional studies on clinical samples. However, it is important to clarify that children with DLD who were not classified as having dyslexia were nonetheless below age expectation in decoding skills (*d *=* *−1.10 relative to TD controls at *t*5). A similar pattern can be seen in spelling, with the DLD group showing smaller impairments than the dyslexia groups (as their phonological skills improve) but still moderate to large deficits relative to controls, consistent with Bishop et al. (2009).

Finally, our findings reveal that the difficulties of children with DLD include deficits in motor and executive skills (e.g., Henry, Messer, & Nash, 2012). In contrast, children with dyslexia‐only perform similar to TD controls, although it is clear from the mean scores that some individuals in the group are impaired. The status of the motor and executive deficits as “risk” factors for DLD or dyslexia is unclear. Neither set of skills is a strong correlate of reading, and this is borne out by the finding that there is no difference between the children with dyslexia + DLD and those with DLD‐only on tasks tapping motor skills or executive function. Within the framework proposed by Barkley (1997), “inner speech” is important for the development of behavioral regulation (see also Bono & Bizri, 2014). It follows that children with language difficulties will experience problems with a range of executive skills. However, evidence suggests that language and executive skills, though strongly correlated, develop independently of one another (Gooch, Hulme, Nash, & Snowling, 2014). In this light, our preferred interpretation is that deficits in executive functions (and plausibly motor skills) are a marker of wider neural deficits, rather than a specific predictor of either language or literacy. This does not rule out the possibility within the multiple‐risk framework (Pennington, 2006) that children with poorer attention or executive skills have fewer resources to bring to the task of learning to read and hence are more likely to experience reading failure.

The present findings suggest that the Bishop and Snowling (2004) framework of the relationship between dyslexia and DLD needs modification to take account of developmental change: Whether or not dyslexia and DLD are regarded as comorbid conditions depends crucially upon the age and stage of development reached. Although the present study corroborates the view that dyslexia and DLD share phonological deficits, it also clarifies that the severity of these and how these develop over time can moderate literacy outcome; it is only when phonological deficits persist that the outcome is dyslexia.

An outstanding question is how differences in the developmental trajectory of DLD‐only and DLD + dyslexia can be interpreted. An obvious possibility is that the improvement in phonological skills of the DLD‐only group is a consequence of learning to read (Castles & Coltheart, 2004). However, this begs the question of how these children learn to read in the face of concurrent phonological difficulties. It may be that the children who learn to read more easily have less severe phonological difficulties (the severity hypothesis); alternatively, they might have had better literacy teaching. Our data are not rich enough to address this possibility. However, it is notable that, although these two groups do not differ in SES, their parents differ in literacy attainments and, it can be assumed, in their own literacy practices (Puglisi, Hulme, Hamilton, & Snowling, 2017). Hence, there are likely genetic differences underpinning the different developmental trajectories as well as differences in gene–environment correlation that have indirect effects on reading development.

### Conclusions

Using a large longitudinal data set, the present study extends previous research by showing that phonological deficits are shared risk factors for dyslexia and DLD. In dyslexia, these are relatively specific, whereas in DLD they occur in the context of poor language and weak nonverbal skills. Emergent literacy deficits in dyslexia observed in preschool become entrenched in the profile shown in the early school years and associated with large deficits in letter knowledge, PA, RAN, decoding, and spelling. For those with comorbid dyslexia + DLD, oral language problems extend beyond phonology, and impairments in reading and PA are generally more severe than those observed in dyslexia or DLD without dyslexia. Children with DLD, with and without dyslexia, have long‐standing broad language deficits as well as deficits in executive and motor skills, the causal status of which is unknown.

## Supporting information


**Table S1.** Constructs Measured at *t*1–*t*5 Showing Measures Analyzed in this Study
**Table S2.** Performance on Language and Literacy Measures From *t*1 to *t*5
**Table S3.** Performance on Measures of Motor and Executive Skills

**Appendix S1.** Details of recruitment, ascertainment criteria and assessment tests

## References

[cdev13216-bib-0001] Arnett, A. B. , Pennington, B. F. , Peterson, R. L. , Willcutt, E. G. , DeFries, J. C. , & Olson, R. K. (2017). Explaining the sex difference in dyslexia. Journal of Child Psychology and Psychiatry, 58, 719–727. 10.1111/jcpp.12691 28176347 PMC5438271

[cdev13216-bib-0002] Barkley, R. A. (1997). Behavioral inhibition, sustained attention, and executive functions: Constructing a unifying theory of ADHD. Psychological Bulletin, 121(1), 65–94. 10.1037/0033-2909.121.1.65 9000892

[cdev13216-bib-0003] Bishop, D. V. M. (2003). The Test for Reception of Grammar (TROG–2). London, UK: Psychological Corporation.

[cdev13216-bib-0004] Bishop, D. V. M. , & Adams, C. (1990). A prospective study of the relationship between specific language impairment, phonological disorders and reading retardation. Journal of Child Psychology and Psychiatry, 31, 1027–1050. 10.1111/j.1469-7610.1990.tb00844.x 2289942

[cdev13216-bib-0006] Bishop, D. V. M. , McDonald, D. , Bird, S. , & Hayiou‐Thomas, M. E. (2009). Children who read words accurately despite language impairment: Who are they and how do they do it? Child Development, 80, 593–605. 10.1111/j.1467-8624.2009.01281.x 19467013 PMC2805876

[cdev13216-bib-0007] Bishop, D. V. M. , & Snowling, M. J. (2004). Developmental dyslexia and specific language impairment: Same or different? Psychological Bulletin, 130, 858–888. 10.1037/0033-2909.130.6.858 15535741

[cdev13216-bib-0008] Bishop, D. V. M. , Snowling, M. J. , Thompson, P. A. , Greenhalgh, T. ; CATALISE‐2 Consortium . (2017). CATALISE: A multinational and multidisciplinary Delphi consensus study of problems with language development. Phase 2. Terminology. Journal of Child Psychology & Psychiatry, 58, 1068–1080. 10.1111/jcpp.12721 28369935 PMC5638113

[cdev13216-bib-0009] Bono, K. E. , & Bizri, R. (2014). The role of language and private speech in preschoolers’ self‐regulation. Early Child Development and Care, 184, 658–670. 10.1080/03004430.2013.813846

[cdev13216-bib-0010] Breckenridge, K. (2008, August). Attention and executive function in Williams syndrome and Down's syndrome. Paper presented at the Development of Executive Functions Workshop [Apples task], The University of Oxford, Oxford, UK.

[cdev13216-bib-0011] Brownell, R. (2000). Expressive and receptive one‐word picture vocabulary tests: Manual (2nd ed.). Novato, CA: Academic Therapy.

[cdev13216-bib-0012] Burrage, M. S. , Ponitz, C. C. , McCready, E. A. , Shah, P. , Sims, B. C. , & Jewkes, A. M. (2008). Age‐ and schooling‐related effects on executive functions in young children: A natural experiment. Child Neuropsychology, 14, 510–524. 10.1080/09297040701756917 18982508

[cdev13216-bib-0013] Carroll, J. , & Snowling, M. J. (2001). The effects of global similarity between stimuli on children's judgment of rime and alliteration. Applied Psycholinguistics, 22, 327–342. Retrieved from https://www.cambridge.org/core/journals/applied-psycholinguistics/article/the-effects-of-global-similarity-between-stimuli-on-childrens-judgment-of-rime-and-alliteration/841E816015AF665991298BF6A0ED6B50

[cdev13216-bib-0014] Carroll, J. M. , Solity, J. , & Shapiro, L. R. (2016). Predicting dyslexia using prereading skills: The role of sensorimotor and cognitive abilities. Journal of Child Psychology and Psychiatry, 57, 750–758. 10.1111/jcpp.12488 26662375 PMC4991277

[cdev13216-bib-0015] Castles, A. , & Coltheart, M. (2004). Is there a causal link from phonological awareness to success in learning to read? Cognition, 91, 77–111. 10.1016/S0010-0277(03),00164-1 14711492

[cdev13216-bib-0016] Catts, H. W. , Adlof, S. M. , Hogan, T. P. , & Weismer, S. E. (2005). Are specific language impairment and dyslexia distinct disorders? Journal of Speech, Language, and Hearing Research, 48, 1378–1396. 10.1044/1092-4388(2005/096) PMC2853030 16478378

[cdev13216-bib-0017] Catts, H. W. , Fey, M. E. , Tomblin, J. B. , & Zhand, X. (2002). A longitudinal investigation of reading outcomes in children with language impairments. Journal of Speech, Hearing & Language Research, 45, 1142–1157. 10.1044/1092-4388(2002/093) 12546484

[cdev13216-bib-0018] Dodd, B. , Hua, Z. , Crosbie, S. , Holm, A. , & Ozanne, A. (2002). Diagnostic Evaluation of Articulation and Phonology (DEAP). London, UK: Psychology Corporation.

[cdev13216-bib-0019] Forum for Research into Language and Literacy . (2012). Diagnostic test of word reading processes. London, UK: GL Assessment.

[cdev13216-bib-0020] Foster, H. (2007). Single Word Reading Test 6–16. London, UK: GL Assessment.

[cdev13216-bib-0021] Gathercole, S. E. , & Baddeley, A. D. (1990). The role of phonological memory in vocabulary acquisition: A study of young children learning arbitrary names of toys. British Journal of Psychology, 81, 439–454. 10.1111/j.2044-8295.1990.tb02371.x

[cdev13216-bib-0022] Gooch, D. C. , Hulme, C. , Nash, H. M. , & Snowling, M. J. (2014). Comorbidities in preschool children at‐risk of dyslexia: The role of language ability. Journal of Child Psychology and Psychiatry, 55, 237–246. 10.1111/jcpp.12139 24117483 PMC4523595

[cdev13216-bib-0023] Henderson, S. E. , Sugden, D. A. , & Barnett, A. L. (2007). Movement Assessment Battery for Children (ABC–2) (2nd ed.). London, UK: Harcourt Assessment.

[cdev13216-bib-0024] Henry, L. A. , Messer, D. J. , & Nash, G. (2012). Executive functioning in children with specific language impairment. Journal of Child Psychology and Psychiatry, 53, 37–45. 10.1111/j.1469-7610.2011.02430.x 21668446

[cdev13216-bib-0025] Hulme, C. , Nash, H. M. , Gooch, D. , Lervag, A. , & Snowling, M. J. (2015). The foundations of literacy development in children at familial risk of dyslexia. Psychological Science, 26, 1877–1886. 10.1177/0956797615603702 26525072 PMC4676358

[cdev13216-bib-0026] Hulme, C. , Stothard, S. E. , Clarke, P. , Bowyer‐Crane, C. , Harrington, A. , Truelove, E. , & Snowling, M. J. (2009). YARC York Assessment of Reading for Comprehension. Early reading. London, UK: GL Assessment.

[cdev13216-bib-0027] Leppänen, P. H. T. , Hämäläinen, J. A. , Guttorm, T. K. , Eklund, K. M. , Salminen, H. , Tanskanen, A. , … Lyytinen, H. (2011). Infant brain responses associated with reading‐related skills before school and at school age. Clinical Neurophysiology, 42, 35–41. 10.1016/j.neucli.2011.08.005 22200340

[cdev13216-bib-0028] McArthur, G. M. , Hogben, J. H. , Edwards, V. T. , Heath, S. M. , & Mengler, E. D. (2000). On the “specifics” of specific reading disability and specific language impairment. Journal of Child Psychology and Child Psychiatry, 41, 869–874. 10.1111/1469-7610.00674 11079429

[cdev13216-bib-0029] Nash, H. M. , Hulme, C. , Gooch, D. , & Snowling, M. J. (2013). Preschool language profiles of children at family risk of dyslexia: Continuities with SLI. Journal of Child Psychology & Psychiatry, 54, 958–968. 10.1111/jcpp.12091 23772651 PMC4523580

[cdev13216-bib-0030] Nation, K. (2005). Children's reading comprehension difficulties In Snowling M. J. & Hulme C. (Eds.), The science of reading (pp. 248–265). Oxford, UK: Blackwell.

[cdev13216-bib-0031] Pennington, B. F. (2006). From single to multiple deficit models of developmental disorders. Cognition, 101, 385–413. 10.1016/j.cognition.2006.04.008 16844106

[cdev13216-bib-0032] Pennington, B. F. , & Bishop, D. (2009). Relations among speech, language, and reading disorders. Annual Review of Psychology, 60, 283–306. 10.1146/annurev.psych.60.110707.163548 18652545

[cdev13216-bib-0033] Pennington, B. F. , Santerre‐Lemmon, L. , Rosenberg, J. , MacDonald, B. , Boada, R. , Friend, A. , … Olson, R. K. (2012). Individual prediction of dyslexia by single versus multiple deficit models. Journal of Abnormal Psychology, 121, 212–224. 10.1037/a0025823 22022952 PMC3270218

[cdev13216-bib-0034] Pickering, S. J. , & Gathercole, S. E. (2001). Working Memory Test Battery for Children (WMTB‐C). London, UK: Psychological Corporation.

[cdev13216-bib-0035] Puglisi, M. , Hulme, C. , Hamilton, L. , & Snowling, M. J. (2017). The home literacy environment is a correlate, but perhaps not a cause, of variations in children's language and literacy development. Scientific Studies of Reading, 21, 498–514. 10.1080/10888438.2017.1346660 29930486 PMC5972965

[cdev13216-bib-0036] Ramus, F. , Marshall, C. R. , Rosen, S. , & van der Lely, H. K. J. (2013). Phonological deficits in specific language impairment and developmental dyslexia: Towards a multidimensional model. Brain, 136, 630–645. 10.1093/brain/aws356 23413264 PMC3572935

[cdev13216-bib-0037] Rice, M. L. , & Wexler, K. (2001). Rice/Wexler Test of Early Grammatical Impairment (TEGI). San Antonio, TX: The Psychological Corporation.

[cdev13216-bib-0038] Rutter, M. , Caspi, A. , Fergusson, D. , Horwood, L. J. , Goodman, R. , Maughan, B. , … Carroll, J. (2004). Sex differences in developmental reading disability: New findings from 4 epidemiological studies. Journal of the American Medical Association, 291, 2007–2012. 10.1001/jama.291.16.2007 15113820

[cdev13216-bib-0039] Scarborough, H. S. (1990). Very early language deficits in dyslexic children. Child Development, 61, 1728–1743. 10.1111/j.1467-8624.1990.tb03562.x 2083495

[cdev13216-bib-0040] Seeff‐Gabriel, B. , Chiat, S. , & Roy, P. (2008). The early repetition battery. London, UK: Pearson Assessment.

[cdev13216-bib-0041] Semel, E. , Wiig, E. H. , & Secord, W. (2006). Clinical Evaluation of Language Fundamentals–fourth edition UK (CELF–4UK). London, UK: Harcourt Assessment.

[cdev13216-bib-0042] Snowling, M. J. (2008). Specific disorders and broader phenotypes: The case of dyslexia. Quarterly Journal of Experimental Psychology, 61, 142–156. 10.1080/17470210701508830 18038345

[cdev13216-bib-0043] Snowling, M. J. , Dawes, P. , Nash, H. , & Hulme, C. (2012). Validity of a protocol for adult self‐report of dyslexia and related difficulties. Dyslexia, 18, 1–15. 10.1002/dys.1432 22271419 PMC3382192

[cdev13216-bib-0044] Snowling, M. J. , Duff, F. J. , Nash, H. M. , & Hulme, C. (2016). Language profiles and literacy outcomes of children with resolving, emerging, or persisting language impairments. Journal of Child Psychology and Psychiatry, 57, 1360–1369. 10.1111/jcpp.12497 26681150 PMC5132029

[cdev13216-bib-0045] Snowling, M. J. , & Hulme, C. (2012). Interventions for children's language and literacy difficulties. International Journal of Disorders of Language & Communication, 47(1), 27–34. 10.1111/j.1460-6984.2011.00081 PMC3429860 22268899

[cdev13216-bib-0046] Snowling, M. J. , & Melby‐Lervåg, M. (2016). Oral language deficits in familial dyslexia: A meta‐analysis and review. Psychological Bulletin, 142, 498–545. 10.1037/bul0000037 26727308 PMC4824243

[cdev13216-bib-0048] Stanovich, K. E. (1994). Does dyslexia exist? Journal of Child Psychology and Psychiatry, 35, 579–595. 10.1111/j.1469-7610.1994.tb01208.x 8040216

[cdev13216-bib-0049] Stanovich, K. E. , Siegel, L. S. , & Gottardo, A. (1997). Progress in the search for dyslexia subtypes In Hulme C. & Snowling M. (Eds.), Dyslexia: Biology, cognition, and intervention (pp. 108–130). London, UK: Whurr.

[cdev13216-bib-0050] Torppa, M. , Lyytinen, P. , Erskine, J. , Eklund, K. , & Lyytinen, H. (2010). Language development, literacy skills, and predictive connections to reading in finnish children with and without familial risk for dyslexia. Journal of Learning Disabilities, 43, 308–321. 10.1177/0022219410369096 20479461

[cdev13216-bib-1000] Office for National Statistics (2010). https://www.ons.gov.uk/census/2001censusandearlier

[cdev13216-bib-0051] van der Leij, A. , van Bergen, E. , van Zuijen, T. , de Jong, P. F. , Maurits, N. , & Maassen, B. (2013). Precursors of developmental dyslexia: An overview of the longitudinal Dutch dyslexia programme study. Dyslexia, 19, 191–213. 10.1002/dys.1463 24133035

[cdev13216-bib-0052] Vellutino, F. R. , Fletcher, J. M. , Snowling, M. J. , & Scanlon, D. M. (2004). Specific reading disability (dyslexia): What have we learned in the past four decades? Journal of Child Psychology & Psychiatry, 45(1), 2–40. 10.1046/j.0021-9630.2003.00305.x 14959801

[cdev13216-bib-0053] Viding, E. , Spinath, F. M. , Price, T. S. , Bishop, D. V. M. , Dale, P. S. , & Plomin, R. (2004). Genetic and environmental influence on language impairment in 4‐year‐old same‐sex and opposite‐sex twins. Journal of Child Psychology and Psychiatry, 45, 315–325. 10.1111/j.1469-7610.2004.00223.x 14982245

[cdev13216-bib-0054] Wechsler, D. (2002). Wechsler Preschool and Primary Scale of Intelligence–third edition (WPPSI–III). San Antonio, TX: Harcourt Assessment.

[cdev13216-bib-0055] Wechsler, D. (2003). Wechsler Intelligence Scale for Children (WISC) (4th ed.). San Antonio, TX: Psychological Corporation.

[cdev13216-bib-0056] Wechsler, D. (2005). Wechsler Individual Achievement Test (WIAT–II) (2nd ed.). San Antonio, TX: Psychological Corporation.

[cdev13216-bib-0500] Wiig, E. H. , Secord, W. , & Semel, E. M. (2004). CELF preschool 2: clinical evaluation of language fundamentals preschool. Pearson/PsychCorp.

[cdev13216-bib-0057] Wiig, E. H. , Semel, E. , & Secord, W. A. (2006). Clinical Evaluation of Language Fundamentals, 4th edition (CELF–4). Bloomington, MN: Pearson.

