# Supplementary material for: Developmental Outcomes for Children at High Risk of Dyslexia and Children With Developmental Language Disorder
Source: Child Dev. 2019 Jan 24;90(5):e548–64. doi: 10.1111/cdev.13216 (PMC6767399; doi:10.1111/cdev.13216)
Supplement: Supplementary file 1 — Table S1. Constructs Measured at t1–t5 Showing Measures Analyzed in this Study Table S2. Performance on Language and Literacy Measures From t1 to t5 Table S3. Performance on Measures of Motor and Executive Skills [file CDEV-90-e548-s001.docx]

*On-Line Appendix [follows]*

Supplementary Table 1

*Constructs Measured at* t*1–*t*5 Showing Measures Analysed in this Study*

|  | **t1** | **t2** | **t3** | **t4** | **t5** |
| --- | --- | --- | --- | --- | --- |
|  | Age = 45 mths.  (SD = 3.5) | Age = 56 mths.  (SD = 3.7) | Age = 68 mths.  (SD = 3.5) | Age = 79 mths.  (SD = 4.4) | Age = 97 mths.  (SD = 5.9) |
| **Nonverbal Ability** | WPPSI Object Assembly, Block Design |  |  |  | WISC IV Block Design, Matrix Reasoning |
| **Language** | CELF Basic Concepts |  |  |  |  |
|  | CELF Sentence Structure | CELF Sentence Structure | CELF Sentence Structure | TROG II | TROG II |
|  | SIT16 | SIT16 & ESIT | ESIT | ESIT | ESIT |
|  | TEGI | TEGI | TEGI | CELF Word Structure | CELF Formulated Sentences |
|  |  | ROWPVT |  |  |  |
|  | CELF Expressive Vocabulary |  | CELF Expressive Vocabulary | CELF Expressive Vocabulary | CELF Expressive Vocabulary |
| **Phonology** | DEAP articulation | DEAP screener | DEAP (only if SSD) |  |  |
|  | PSRep (1–3) | PSRep (1–4) | PSRep nonwords |  |  |
|  |  |  | NNWrep | NNWrep | NNWrep & CNrep |
| **Phonological Awareness** | Syllable matching |  |  |  |  |
|  | Alliteration matching |  |  |  |  |
|  | Phoneme isolation | Phoneme isolation |  |  |  |
|  |  | YARC phoneme deletion | YARC phoneme deletion | YARC phoneme deletion | YARC phoneme deletion |
| **Rapid Naming** |  | RAN objects and colours | RAN objects | RAN objects & digits | RAN objects & digits |
| **Letter Knowledge (GPC)** | Letter knowledge (12) | YARC LSK | YARC LSK/Letter Production | YARC LSK |  |
| **Decoding** |  | YARC EWR | YARC EWR | YARC EWR |  |
|  |  |  | Single Word Reading (SWRT) | Single Word Reading (SWRT) | Single Word Reading (SWRT) |
|  |  |  |  |  | Exception word reading (IWR) |
| **Spelling** |  |  | Spelling early words | Spelling early words (+) | WIAT spelling |
| **Motor Skills** | Drawing shapes |  |  |  |  |
|  | ABC fine motor | ABC fine motor | ABC fine motor | ABC fine motor |  |
|  |  |  |  |  |  |
| **Attention and EF** | Visual search efficiency | Visual search | Visual search | Visual search | Visual search |
|  | HTKS | HTKS | HTKS |  |  |
|  | Go/No-go (dog/bird) |  |  | Go/No-go | Go/No-go |
|  |  | ACPT | ACPT |  |  |
|  |  | Block recall | Block recall | Block recall | Block recall |

Supplementary Table 2

*Performance on Language and Literacy Measures from* t*1 to* t*5*

|  | Typical Reader Outcome – whole sample  (N = 146) | TD Control – no disorder at *t*5  (N = 64)  *Benchmark comparison* | Dyslexia  (N = 21) | | DLD  (N = 38) | | Dyslexia + DLD  (N = 29) | |  |
| --- | --- | --- | --- | --- | --- | --- | --- | --- | --- |
|  |  |  |  | Cohen’s *d* |  | Cohen’s *d* |  | Cohen’s d | F value |
| SES^1^ | 7.23 (2.58) | 7.67 (2.41) _1_ | 6.95 (2.85) _1,2_ | .29 | 5.58 (3.15)_2,3_ | .77 | 5.72 (2.90) _2,3_ | .76 | 5.47 |
| Performance IQ *t*1^2^ | 111.96 (14.14) | 115.81 (13.26) _1_ | 106.95 (14.21)_1,2_ | .66 | 100.08 (14.42) _2_ | 1.15 | 101.32 (11.62)_2_ | 1.14 | 12.92 |
| Language *t*1 | .29 (.65) | .53 (.52) _1_ | .28 (.73)_1_ | .43 | -.83 (.64) _2_ | 2.38 | -.69 (.59) _2_ | 2.24 | 41.81 |
| Language *t*2 | .34 (.60) | .46 (.49) _1_ | .33 (.54) _1_ | .26 | -.82 (.68) _2_ | 2.25 | -.76 (.61) _2_ | 2.30 | 55.67 |
| Language *t*3 | .40 (.48) | .46 (.47) _1_ | .24 (.50) _1_ | .46 | -.79 (.70) _2_ | 2.21 | -.88 (.83) _2_ | 2.20 | 51.25 |
| Language *t*4 | .49 (.52) | .66 (.48) | .15 (.58) _1_ | 1.01 | -.81 (.47) _2_ | 3.09 | -1.21 (.74) _3_ | 3.38 | 100.88 |
| Language *t*5 | .52 (.49) | .60 (.52) | .09 (.50) _1_ | .99 | -1.07 (.46) _2_ | 3.35 | -1.28 (.69) _2_ | 2.82 | 121.78 |
| Phonology *t*1 | .29 (.64) | .56 (.31) _1_ | .24 (.49) _1_ | .75 | -.68 (.99) _2_ | 1.93 | -.46 (.82) _2_ | 2.36 | 29.85 |
| Phonology *t*2 | .32 (.65) | .58 (.25) _1_ | .14 (.61) _1_ | 1.19 | -.70 (1.04) _2_ | 1.93 | -.84 (1.02) _2_ | 2.36 | 36.68 |
| Phonology *t*3 | .35 (.59) | .56 (.69) | -.18 (.75) _1_ | 1.04 | -.42 (.77) _1_ | 1.36 | -.92 (.82) _2_ | 2.02 | 30.34 |
| Phonology *t*4 | .34 (.66) | .55 (.58) | -.01 (.85) _1_ | .85 | -.42 (.76) _1_ | 1.49 | -1.06 (.83) _2_ | 2.41 | 36.23 |
| Phonology *t*5 | .33 (.64) | .56 (.68) | -.17 (.89) _1_ | .97 | -.36 (.74) _1_ | 1.31 | -1.16 (.86) _2_ | 2.32 | 34.04 |
| Phon Aw *t*2 | .23 (.72) | .37 (.63) | -.31 (.66) _1_ | 1.07 | -.66 (.49) _1_ | 1.78 | -.71 (.51) _1_ | 1.82 | 23.22 |
| Phon Aw *t*3 | .34 (.52) | .42 (.49) | -.40 (.70) _1,2_ | 1.50 | -.33 (.76) _1,2_ | 1.24 | -.88 (.79) _1_ | 2.17 | 30.17 |
| Phon Aw *t*4/*t*5 | .39 (.66) | .52 (.65) | -.61 (.64)_1_ | 1.75 | -.28 (.61) _1_ | 1.26 | -1.16 (.54) | 2.72 | 54.92 |
| RAN *t*2 | .27 (.84) | .57 (.84) | -.64 (.70)_1_ | 1.50 | -.56 (.99) _1_ | 1.25 | -.41 (.48) _1_ | 1.32 | 16.93 |
| RAN *t*3 | .31 (.74) | .46 (.77) | -.38 (.62) _1_ | 1.15 | -.46 (.78) _1_ | 1.19 | -.54 (.67) _1_ | 1.37 | 19.62 |
| RAN *t*4 | .30 (.65) | . 45 (.62) | -.52 (.76) _1,2_ | 1.46 | -.27 (.82) _1_ | 1.03 | -.79 (.66) _2_ | 1.95 | 24.72 |
| RAN *t*5 | .29 (.70) | .34 (.59) | -.62 (.68) _1,2_ | 1.60 | -.22 (.70) _1_ | .90 | -.72 (.75) _2_ | 1.67 | 22.77 |
| LK *t*1^3^ | 3.84 (3.70) | 5.00 (3.97) | 2.26 (2.37) _1_ | .76 | 1.24 (1.83) _1_ | 1.14 | 1.11 (1.99) _1_ | 1.13 | 16.95 |
| LK *t*2^4^ | 19.52 (9.17) | 20.73 (8.96) | 13.48 (10.26) _1_ | .78 | 11.05 (7.89) _1_ | 1.13 | 11.5 (10.57) _1_ | .97 | 11.82 |
| LSK *t*3^4^ | 30.81 (1.70) | 31.03 (1.58) | 26.10 (6.51) _1,2_ | 1.42 | 28.21 (3.63) _1,2_ | 1.11 | 23.40 (7.35) _1_ | 1.79 | 21.00 |
| LSK *t*4^4^ | 31.56 (.81) | 31.73 (.89) | 29.81 (2.37) _1_ | 1.52 | 30.97 (2.0) _1_ | .59 | 26.86 (5.45) | 1.59 | 22.06 |
| Decoding *t*2 | .20 (1.03) | .39 (1.17) | -.27 (.32) _1_ | .64 | -.37 (.16) _1_ | .81 | -.38 (.14) _1_ | .79 | 11.02 |
| Decoding *t*3 | .44 (.90) | .61 (.88) | -.65 (.41) _1_ | 1.59 | -.54 (.62) _1_ | 1.45 | -.91 (.25) _1_ | 2.04 | 46.31 |
| Decoding *t*4 | .47 (.61) | .64 (.44) | -.91 (.65) _1_ | 3.11 | -.23 (.71) | 1.55 | -1.19 (.66) _1_ | 3.53 | 79.26 |
| Decoding *t*5 | .56 (.57) | .56 (.53) | -1.16 (.75) _1_ | 2.90 | -.08 (.66) | 1.10 | -1.49 (.84) _1_ | 3.18 | 76.73 |
| Spelling *t*3^5^ | 2.57 (1.13) | 2.71 (1.11) | 1.14 (.96) _1,2_ | 1.46 | 1.24 (1.10) _1,2_ | 1.33 | .48 (.83) _1_ | 2.16 | 37.87 |
| Spelling *t*4^6^ | 6.16 (2.64) | 6.52 (2.61) | 2.71 (1.27) _1_ | 1.62 | 4.42 (2.07) | .87 | 1.72 (1.44) _1_ | 2.07 | 39.84 |
| Spelling *t*5^7^ | 27.88 (5.35) | 28.38 (5.56) | 18.05 (2.8)_1_ | 2.05 | 23.21 (4.42) | 1.00 | 16.69 (5.22) _1_ | 2.14 | 48.04 |

*Table Notes:*^1^postcode rating (at *t*2); ^2^standard score; ^3^max =12; ^4^max = 32; ^5^max = 5; ^6^max =10; ^7^WIAT raw score

Supplementary Table 3

*Performance on Measures of Motor and Executive Skills*

|  | Typical Reader  Whole sample  (N = 146) | TD-control (no NDD)  *Benchmark comparison*  (N = 64) | Dyslexia  (N = 21) | | DLD  (N = 38) | | Dyslexia + DLD  (N = 29) | |  |
| --- | --- | --- | --- | --- | --- | --- | --- | --- | --- |
|  |  |  |  | Cohen’s *d* |  | Cohen’s *d* |  | Cohen’s *d* | F value |
| Motor *t*1 | -.21 (.66) | -.33 (.57) _1_ | -.21 (.73) _1,2_ | .20 | .47 (.93) _3_ | 1.12 | .21 (.97) _2,3_ | .79 | 7.95 |
| Motor *t*2 | -.27 (.63) | -.27 (.52) _1_ | .05 (.84) _1, 2_ | .52 | .50 (.95) _1,2_ | 1.09 | .50 (1.16) _1,2_ | 1.00 | 9.05 |
| Motor *t*3 | -.20 (.67) | -.15 (.69) _1_ | .09 (.66) _1,2_ | .35 | .30 (.88) _1,2_ | .59 | .48 (1.18) _2_ | .73 | 4.56 |
| Motor *t*4 | -.18 (.63) | -.29 (.60) _1_ | -.12 (.90) _1,2_ | .25 | .29 (.86) _2,3_ | .82 | .53 (1.10) _3_ | 1.04 | 8.11 |
| EF *t*2 | .24 (.66) | .39 (.49) | .18 (.55) _1_ | .83 | -.32 (.41) _1_ | 1.46 | -.45 (.28) _1_ | 1.43 | 17.42 |
| EF *t*3 | .28 (.49) | .30 (.54) _1_ | -.01 (.54) _1,2_ | .49 | -.46 (.62) _2,3_ | 1.19 | -.56 (.82) _3_ | 1.22 | 17.71 |
| EF *t*4 | .21 (.48) | .20 (.51) _1_ | -.02 (.59) _1,2_ | .35 | -.34 (.64) _2,3_ | .96 | -.50 (.72)_3_ | 1.21 | 11.67 |
| EF *t*5 | .15 (.47) | .16 (.45) _1_ | -.02 (.51) _1,2_ | .34 | -.38 (.56) _2_ | .96 | -.28 (.84) _2_ | 1.20 | 8.34 |
