# Supplementary material for: Developmental Outcomes for Children at High Risk of Dyslexia and Children With Developmental Language Disorder
Source: Child Dev. 2019 Jan 24;90(5):e548–64. doi: 10.1111/cdev.13216 (PMC6767399; doi:10.1111/cdev.13216)
Supplement: Supplementary file 2 — Appendix S1. Details of recruitment, ascertainment criteria and assessment tests [file CDEV-90-e548-s002.docx]

**Recruitment and Ascertainment Criteria**

(taken from published material)

*Participants*

Families were recruited to the study via advertisements placed in local newspapers, nurseries and the webpages of support agencies for children with reading and language difficulties and via speech and language therapy services. Of the 242 children recruited, none met our exclusionary criteria (MZ twinning, chronic illness, deafness, English as a 2^nd^ language, care provision by local authority and known neurological disorder such as cerebral palsy, epilepsy, ASD).

Following recruitment, children were classified using a two-stage process: first we determined whether they were at FR of dyslexia and then diagnostic criteria were used to ascertain whether they had a language impairment (SLI). This led to the classification of children into four groups: FR-only; FR-SLI; SLI-only, Typically Developing (TD) (See Appendix 1 for full details).

*Family risk.*

Previous FR studies have either used self-report or objective measures to determine risk status. In the current study we obtained self-report using the Adult Reading Questionnaire (ARQ; xxx) and conducted objective testing of parents who consented. To avoid missing children at FR of dyslexia we employed the following criteria: A child was included in the FR group if 1) a parent reported as dyslexic, 2) a parent scored below 90 on a literacy composite of nonword reading and spelling, 3) a parent had a discrepancy between nonverbal ability and the literacy composite of 1.5 standard deviations, with a literacy composite standard score of 96 or below, or 4) a sibling had a diagnosis of dyslexia from an educational psychologist or a specialist teacher. According to these criteria 120 of the 242 children were considered at FR of dyslexia.

*Language impairment.*

LI status was determined using three subtests of the Clinical Evaluation of Language Fundamentals – Preschool 2 UK (CELF-Preschool 2 UK, Semel, Wiig & Secord., 2006); Basic Concepts, Expressive Vocabulary and Sentence Structure and the screener from the Test of Early Grammatical Impairment (TEGI, Rice & Wexler, 2001). These tests assess receptive and expressive language across multiple domains of language (Tomblin et al, 1997). Children were deemed language impaired if they ‘failed’ 2/4 of these language tests (a fail being a scaled score of 7 or below on the CELF subtests and failure of the TEGI screener). Given the age and low ability of some of the children there were insufficient data from the diagnostic tests to determine LI status for 22 cases. In these cases information was used from the separate TEGI screener subtests and the Preschool Repetition test (Seeff-Gabriel, Chiat & Roy., 2008) at T1 and CELF sentence structure test at T2 to come to a clinical judgement about group membership. Seventeen of the 22 cases were considered language impaired.

Based on our diagnostic criteria 35/120 children at FR and 32/46 children referred for speech and language difficulties were language impaired; 74% of these children were in contact with speech and language therapy services at T1. The remaining 14 children whose parents had reported concerns but who did meet our diagnostic criteria were excluded from further analyses. In addition, of the 76 children initially classified as typically developing, 5 met criteria for language impairment. These children were considered to be language impaired for the purposes of this study. Among LI children, 6 had nonverbal IQ (NVIQ) scores below 80 (WPPSI-III; Wechsler, 2003) and a further 3 were not able to complete the tests. Therefore, the majority met criteria for SLI. All children who met LI criteria, regardless of NVIQ were included because research suggests that children in the lower range of NVIQ show the same overall profile of Language Impairment as children in the range of 85 and above (Tomblin & Zhang, 1999). Given that the majority of LI children had NVIQ scores above 80, we will refer to them as SLI. Between T1 and T2 there was a small amount of attrition, 2 TD, 8 FR and 4 SLI children withdrew from the study. Their data are not included here. The following analyses are based on children in four groups, TD (N= 69), FR-only (N=83), SLI (N=32) and FR-SLI (N=29).

The four groups did not differ in age at T1 with averages ranging from 3 years 7 months to 3 years 9 months. There was a non-significant trend for more boys than girls to reach criteria for language impairment. The TD group were higher in SES than the two language impaired groups and the TD group performed significantly better on the NVIQ tasks than the FR group, who in turn performed better than the two language impaired groups.

**Details of Assessment Tests**

***Nonverbal Ability.***

*Wechsler Preschool and Primary Scales of Intelligence (WPPSI-III*; Wechsler, 2003) Block Design and Object Assembly. In Block Design, designs have to be constructed using 3D blocks to match 2-D templates. In Object Assembly the child puts together pieces of a ‘jigsaw-like’ puzzle without a template. Split-half reliability Block Design: = .84; Object Assembly = .85.

*Wechsler Intelligence Scale for Children (WISC-IV*, Wechsler, 2003) Block Design (as above) and Matrix Reasoning in which the child solves a series of nonverbal problems in which a missing detail/piece has to be supplied. Split-half reliability Block Design = .89; Matrix Reasoning = .89.

***Language Measures***

1. Comprehension:
2. *Basic Concepts (CELF-Preschool 2 UK;* Wiig et al, 2006).

The child hears a sentence (e.g. point to the one that is long) and has to select from a choice of three, the picture that demonstrates the concept.

1. Receptive Grammar:
2. *Sentence Structure* (*CELF-Preschool, Wiig et al, 2006; CELF-4,* Semel et al. 2003*)*

The child hears a sentence (e.g. the bear is in the wagon) and has to select from a choice of four, the picture that conveyed its meaning. The sentences included a range of different syntactic structures. (CELF Preschool α=.83;CELF 4 α=.88)

1. *Test for the Reception of Grammar* (*TROG-II* – Bishop, 2003)

The child hears a sentence (e.g. the bear is in the wagon) and has to select from a choice of four, the picture that conveys its meaning. The sentences included a range of different syntactic structures. (TROG 2 α=.88 )

1. Expressive Grammar:
2. *Sentence Imitation Test* (*SIT-16; Seeff-Gabriel, Chiat, Roy, 2008*)

The child repeats 16 sentences increasing in length and complexity (e.g. the cat ate a big mouse). The total number of sentences, content words, function words and grammatical inflections repeated correctly was recorded. (α=.92)

1. *Experimental Sentence Imitation Test*, a test designed for the study. The child repeats 20 sentences: 10 (5 long/5 short) containing transitive verbs and 10 (5 long/5 short) containing ditransitive verbs. The measure is number of sentences correctly repeated (α=.78).
2. *Test of Grammatical Inflections (TEGI; Rice & Wexler, 2001)*.

The third person and past tense probes, together provide a screening test score reflecting the ability to produce grammatical inflections. In the third person singular /s/ probe (10 items) the child is introduced to characters and asked to describe what each does. For example, the child is shown a picture of a dentist and asked to say what a dentist does. A response containing the third person singular /s/ inflection is scored as correct (e.g. a dentist checks / cleans your teeth). (split-half *r* = .92) In the past tense probe, the child is shown two pictures, the first picture described by the examiner (e.g. here the boy is brushing his hair). The examiner then points to the second picture and says ‘now he is done, tell me what he did’. (split-half *r* = .82)

Correctly inflected verbs (e.g. brushed / combed) and over-regularisations of irregular verbs are scored as correct. In both subtests the score is the percentage of items correct (non-responses and other verb tenses were excluded and only bare stems were scored as incorrect). In order to correctly inflect the nouns and verbs in these subtests the child needs to be able to produce /s/, /z/, /t/ and /d/.

1. *Word Structure* (*CELF-4 op cit)*

The child is shown a picture and has to say something about it. The instructions are: I’m going to show you some pictures and say some things about them. I want you to help me by finishing some of the things I say”. There are 3 practice items in which support can be given before the test items, as detailed in the manual:

1: Here is a boy [point] and here [point]­_______ (*is a girl*)

2: This boy [point] is standing and this boy [point] _______ (*is sitting*)

3: Lee [point to young girl on the left] said, “Those shoes are yours and these shoes_______” (*are mine*)

Responses are score 1 (correct) or 0 (incorrect). (α=.78-.86)

1. *Formulated Sentences* (*CELF-4 op cit)*

The child is shown a picture and has to use a target word or words to produce a sentence about the picture. Following a demonstration and two practice items which are given with corrective feedback before 28 test items. For example: “Here is a picture. Now you make a sentence about this picture using the word ***playing.*** You must make you your sentence about something in the picture and you must use the word ***playing****.* Look at the picture to help you think of what to say.” If the child requests a repetition or produces a phrase/sentence that does not include the target word or is not appropriate to the stimulus picture then the instructions are repeated to remind them to use the target word and to make the sentence about the picture. Responses to test items are scored 0,1 or 2 according to the test manual. The test is discontinued after 5 items receive a score of 0. (α=.76)

1. Vocabulary:
2. *Expressive Vocabulary* test (*CELF-Preschool 2 UK, Wiig et al, 2006; CELF-4 (*Semel et al. 2003)

The child names a series of pictures (objects, e.g. carrot, telescope) or describe what a person is doing (e.g. riding a bike) (α=.84) The test was extended with a further 8 items from the *Expressive One Word Picture Vocabulary Test* (Brownell, 2000) to guard against ceiling effects at t4, t5 (hammock, funnel, scroll, anvil, tripod, protractor, observatory, metronome) (α=.66)

1. *Receptive One Word Picture Vocabulary Test (ROWPVT; Brownell, 2000*).

The child heard a word and was asked to select the corresponding picture, from a choice of four (α =.95).

***Phonology Measures***

1. S*peech:*
2. *Diagnostic Evaluation of Articulation and Phonology (DEAP*, Dodd, Hua, Crosbie, Holm & Ozanna, 2002). The articulation subtest requires the child to name 30 pictures (e.g. pig, moon, sheep, five, television) or imitate the name if they cannot produce it spontaneously. The percentage of consonants correctly produced is recorded.

*Repetition:*

*Early Repetition Battery* (Seeff-Gabriel, Chiat, Roy, 2008*)* The child is asked to repeat 18 words (6 one-syllable, 6 two-syllable and 6 three-syllable) and 18 nonwords (6 of each length). The nonwords are created from the words, by altering the vowel in the one-syllable items and by swapping two consonants in the multi-syllabic items (e.g., ‘lamb’ -> /lom/, ‘machine’ -> /shameen/, ‘dinosaur’ -> /sinodaur/). (α=.89)

At t2 of the present study, the repetition tests were extended to include 6 additional 4 syllable words /nonwords to avoid ceiling effects.

*New Nonword Repetition (NNWRep)*

The child repeats nonwords of 2-4 syllables in length varying in wordlikeness (low versus high). At each syllable length there were 5 low and 5 high word-like items. The number of nonwords repeated correctly is measured at each syllable length and a total score recorded. (αs=.77-.80)

*Children’s Nonword Repetition Test (CNRep*: Gathercole & Baddeley, 1990)

The child repeats nonwords of 2-4 syllables, 10 at each syllable length (e.g. *blonterstaping*). The number repeated correctly is measured at each syllable length and a total score recorded. (Test-retest *r* = .77; split-half *r* = .66).

***Phonological Awareness***

1. *Syllable matching*  (from Carroll, Hulme, Snowling & Stevenson, 2003; *Developmental Psychology*, *39*(5), 913.

At the start of the task, the children are introduced to a puppet, Gerry Giraffe, who likes to collect words that start with the same syllable. For each trial, Gerry holds a picture card, and the child is asked, for instance, “Gerry has a picture of *butter*. Which of these words, *sandwich* or *button*, has the same sound at the beginning as *butter*?” When they have chosen, the cards are turned over to see if they are correct - the correct alternative has a coloured sticker on the back that is the same colour as the cue card. The distractor card had a differently coloured sticker. If they have picked correctly, the experimenter says, for instance, “Yes, that’s right. *Butter* and *button* have the same sound, ‘*but*’, at the beginning. *Sandwich* is the odd one out.” If they have chosen the wrong alternative, they are told “No, *button* and *butter* have the same sound, ‘*but*’, at the beginning. *Sandwich* is the odd one out.” In this way, the children are given immediate feedback after every trial. In the final syllable matching trials the procedure is the same except that the puppet is Roger Badger, who likes to collect words that had the same final syllable.

The task begins each time with two compound words and then uses two-syllable words, with two exceptions (television and telephone for the initial syllable condition).

1. *Alliteration Matching (op cit)*

The task paralleled the syllable matching task except this time This time the puppet used was Carrie Cow, who liked to collect words with the same initial sound. As before, feedback was given after each trial. The words for the alliteration matching task were one syllable CVC words: mouth, nut, beach, dish and bean. Four of the distractors were semantically related to the cue word, four were matched for global similarity, and four were both matched for global similarity and were semantically related to the cue word.

1. *Phoneme Isolation*:

The child is asked to repeat a spoken nonword and then to say its first sound. There are 2 demonstration and 2 practice items followed by 8 monosyllabic test items (4 CVC and 4 CCVC). Testing is discontinued after 4 incorrect responses. (α=.71)

Following the initial isolation task, the child is asked to say the last sound in each nonword. There are 2 practice and 8 test items, 4 CVC and 4 CVCC. Testing is discontinued after 4 incorrect responses. (α=.93).

1. Phoneme Deletion (*YARC Early Reading;* Hulme et al., 2009*)*

The child hears a word, repeats it and then says it dropping a specified phoneme (e.g., ‘without the /b/’) (12 items). (α=.95)

At t4 and t5 of the present study, 12 items were added to extend the test (5 words with picture support and 7 nonwords without picture support) to guard against ceiling effects.

***Rapid Automatized Naming***

In the RAN tasks, children are first asked to name each of the RAN stimuli (objects, colours, digits) to check that they know the names. They are then presented with an 8 x 5 array of stimuli (each of the 5 stimuli was presented 8 times in a random order) and told to name each of the stimuli (moving from left to right) as quickly as possible. The time taken to name all 40 stimuli and the number of errors made are recorded. RAN rate is calculated (number correct (max 40)/time (s)).

1. RAN Colours. Children name an 8 x 5 array of 40 stimuli comprising 5 colours (squares coloured brown, blue, black, red and green) as quickly as possible for two trials each. The time taken is measured. (α=.78)
2. RAN Objects. Children name an 8 x 5 array of 40 stimuli comprising 5 objects (pictures of a dog, eye, key, lion and table) as quickly as possible for two trials each. The time taken is measured. (α=.72)
3. RAN Digits. Children name an 8 x 5 array of 40 stimuli comprising 5 digits (2, 1, 5, 8, 4) as quickly as possible for two trials each. The time taken is measured (reliability = .75)

***Literacy Skills***

1. Letter Knowledge: *(York Assessment of Reading and Comprehension; YARC,* Hulme et al., 2009)*.*

The child is asked to give the sound of 12 letters (α=.95) at t1 and later, the sound of 32 single letters and digraphs (α=.92).

1. Decoding Skills
   1. Early Word Reading (*YARC* ; Hulme et al., 2009) . The child reads aloud 30 single words, graded in difficulty. Half of the words are phonemically regular (decodable), and half are irregular. Each correct response scores 1 point; testing is discontinued if the child makes 10 consecutive reading errors.
   2. Single word reading (S*WRT;* Foster, 2007)

The child reads 60 words of increasing difficulty. Testing is discontinued after 5 consecutive errors/refusals (α=.98)

1. *Diagnostic Test of Word Reading Processes* Exception Words (Forum for Research in Language and Literacy, 2012). The child is asked to read 30 irregular words of 1-3 syllables, e.g. come, giant, treacherous (α=.97).
2. Spelling*:*
3. The child is asked to spell 5 words (dog, cup, tent, book, heart), each represented by a picture. They first name each picture but if they make an error, the examiner provides the name before the child attempts to write the word (α=.53);
4. At *t*4, 12 words were given (α=.86)
5. Wechsler Individual Achievement Test of Spelling (*WIAT*; Wechsler, 2005) requires the child to spell single words to dictation. (α=.96).

***Motor Skills***

1. Fine motor skill and eye-hand coordination:
2. *Posting Coins (Movement Assessment Battery for Children-2* ; Henderson, Sugden, & Barnett, 2007)*.* The child posts 12 coins into a money box as quickly as possible and the time taken with each hand was recorded. Stability between t1 and t2 (*r* = .69 and .70 for preferred and non-preferred hands respectively).
3. *Bead Threading (op cit).* The child threads 6 beads onto a piece of string as quickly as possible and the time taken is recorded. Stability between t1 and t2 (*r* = .67).
4. *Bicycle Trails (op cit).* The child uses their preferred hand to draw a single continuous line following a trail without crossing its boundaries and the number of errors was recorded. Stability between T1 and T2 (*r* = .47).

***Executive Function***

1. Visual Search (the *Apples Task*; Breckenridge, 2008)

This is a measure of selective attention. The child is given 1 minute to search an array to identify targets (18 red apples) whilst ignoring distracters (81 red strawberries and 81 white apples). The number of targets identified and the number of commission errors made (pointing to a distracter; false alarms) is recorded. A visual search efficiency score ((Hits: total targets correctly identified – commission errors)/60 seconds) is calculated; a high score reflects better selective attention. Stability between t2-t3 and t3-t4; *r* = .59 and .49 respectively.

1. Auditory Continuous Performance Test *(ACPT*)

This is at test of Sustained Attention An ACPT similar to those developed by Kerns and Rondeau (1998) and Mahone, Pillion and Hiemenz (2001). Children see an image of a farm and hear four different animal sounds (3 distracters (cow, duck and frog) and 1 target (dog)). The children are asked to press a response button when the dog barks. Each auditory stimulus is presented 30 times in a pseudorandom order. Each stimulus lasts 2500ms with ITIs of 1750ms. Reaction times as well as omission and commission errors are recorded. An ACPT efficiency score ((Hits: total targets correctly identified – commission errors)/120 trials) is calculated; a high score reflects better selective attention.

1. The dog/bird task.

This is a version of the Bear-Dragon Go/No-Go task (Reed, Pien & Rothbart, 1984) and is used to measure behavioural inhibition. Children have to inhibit their natural inclination to follow verbal instructions for motor/hand actions (‘thumbs up’, ‘make a fist’, ‘point’ or ‘wave’) given by two puppets (a dog and a bird). The child is instructed to do what the dog says but to ignore the bird (i.e. inhibit their action). The number of times that the child correctly responds to instructions given by the dog (max 8) or incorrectly followed instructions given by the bird (commission errors; max 8) were recorded. A GoNoGo efficiency score (Hits: number of responses to the dog/total number of responses (responses to dog + bird)) was calculated a high score reflects better behavioural inhibition.

1. Go/No-Go task.

In this computerised task, children complete 80 Go/NoGo trials; on 75% of these trials the go stimulus was presented (bug) and on 25% of the trials the no-go stimulus was presented (ladybird). Prior to the GoNoGo trials children complete 30 go trials to establish the prepotent/automatic response which they then had to try and inhibit on the no-go trials. Children are instructed to press a button as quickly as possible (within 2000ms) when they saw the bug but not when they saw the ladybird. The task lasts for approximately 5 minutes and the number of commission errors made on No-Go trials trials is used as an index of behavioural inhibition.

1. Head, Toes, Knees, Shoulders task *(HTKS;* Burrage et al., 2008):

This is a measure of behavioural regulation*.*  The child has to do the opposite of what the examiner said (e.g. touch their toes if asked to touch their head and vice versa). If the child is able to successfully inhibit on 5/10 trials they go on to complete a further block of 10 harder trials with additional commands (e.g. to touch their shoulders if asked to touch their knees and vice versa). Each correct response receives 2 points, self-corrected responses (partial inhibitions; where the child moved towards the incorrect, intuitive response but demonstrated the correct final response) receive 1 point and incorrect responses receive 0 points (max score = 40). Stability between t2 and t3; *r* = .52.

1. Visual-Spatial Memory

The *Block Recall* task from the Working Memory Test Battery for Children (WMTB-C; Pickering & Gathercole, 2001) is a measure of visuo-spatial memory known to cauae difficulty for children with ADHD (α=.63). The child watches the examiner tap a sequence of blocks on a board and then recalls the sequence by tapping the blocks in the same order. The number of correct trials is recorded (max 52). Test-retest reliability = 0.63.
